# A novel theatre-based behaviour change approach for influencing community uptake of schistosomiasis control measures

**DOI:** 10.1186/s13071-022-05421-5

**Published:** 2022-08-25

**Authors:** May N. Sule, Justina Mosha, Teshome Emana Soboka, Safari M. Kinung’hi, Chrysoula Sfynia, Kamran Rafiq, Alex Dower, Marianne Comparet, Emma Bewley, Teckla Angelo, Feleke Zewge Beshah, Michael R. Templeton

**Affiliations:** 1 Department of Civil and Environmental Engineering, Imperial College London, London, UK; 2 Cranfield Water Science Institute, School of Water, Energy and Environment, Cranfield University, Bedford, UK; 3 School of Geography and the Environment, University of Oxford, Oxford, UK; 4 National Institute for Medical Research, Mwanza Centre, Mwanza, Tanzania; 5 Africa Centre of Excellence for Water Management, College of Natural and Computational Sciences, Addis Ababa University, Addis Ababa, Ethiopia; 6 Acting for Health, London, UK

**Keywords:** Schistosomiasis, Water, Sanitation, WASH, Behaviour change, Theatre, Role-play

## Abstract

**Background:**

Appropriate behaviour change with regard to safe water contact practices will facilitate the elimination of schistosomiasis as a public health concern. Various approaches to effecting this change have been trialled in the field but with limited sustainable outcomes. Our case study assessed the effectiveness of a novel theatre-based behaviour change technique (BCT), in combination with cohort awareness raising and capacity training intervention workshops.

**Methodology:**

Our study was carried out in four rural communities in the Mwanza region of Tanzania and in the semi-urban town of Kemise, Ethiopia. We adapted the Risk, Attitude, Norms, Ability and Self-regulation (RANAS) framework and four phases using a mixed methods approach. Participatory project phase engagement and qualitative formative data were used to guide the design of an acceptable, holistic intervention. Initial baseline (BL) data were collected using quantitative questionnaire surveys with 804 participants in Tanzania and 617 in Ethiopia, followed by the theatre-based BCT and capacity training intervention workshops. A post-intervention (PI) survey was carried out after 6 months, with a participant return rate of 65% in Tanzania and 60% in Ethiopia.

**Results:**

The intervention achieved a significant improvement in the knowledge of schistosomiasis transmission being associated with poorly managed sanitation and risky water contact. Participants in Tanzania increased their uptake of preventive chemotherapy (males: BL, 56%; PI, 73%, females: BL, 43%; PI, 50%). There was a significant increase in the selection of sanitation (Tanzania: BL, 13%; PI, 21%, Ethiopia: BL, 63%; PI, 90%), safe water and avoiding/minimising contact with infested waters as prevention methods in Tanzania and Ethiopia. Some of the participants in Tanzania followed on from the study by building their own latrines.

**Conclusions:**

This study showed that substantial positive behaviour changes in schistosomiasis control can be achieved using theatre-based BCT intervention and disease awareness training. With the appropriate sensitisation, education and stakeholder engagement approaches, community members were more open to minimising risk-associated contact with contaminated water sources and were mobilised to implement preventive measures.

**Graphical Abstract:**

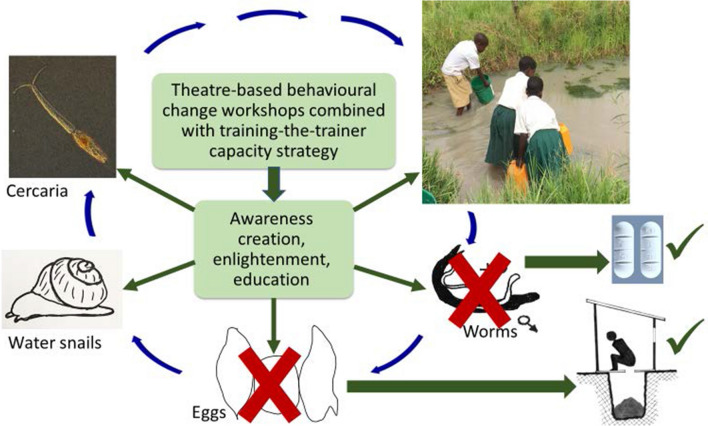

**Supplementary Information:**

The online version contains supplementary material available at 10.1186/s13071-022-05421-5.

## Background

Schistosomiasis affects almost 240 million people worldwide [1], causing 2.5 million disability-adjusted life-years [2] and a significantly high global burden of disease [3]. The WHO endorsed the road map for neglected tropical diseases 2021–2030 [4], with the goal of achieving the elimination of schistosomiasis as a public health problem by 2030, and likewise for other neglected tropical diseases (NTDs). Barriers to achieving the elimination of schistosomiasis include unsafe behaviours regarding domestic, occupational and social water contact practices with contaminated sources [5], inadequate water supply and usage, lack of universal sanitation coverage and hygiene practices [6], partial uptake of mass drug administration (MDA) programmes [7] and lack of uptake of personal protective equipment for occupational water contact [8]. A behaviour change approach can facilitate the desired reduction in contamination of water sources through urine and faeces, change attitudes toward avoiding contaminated water contact and thereby reduce transmission [9].

Changing human behaviours can be a challenging process. It requires a change in contextual (social, physical and personal), environmental and psychosocial factors which are associated with the respective behaviour, such as attitudes, norms and self-regulation, that are already entrenched in an individual or community [10]. Within the water, sanitation and hygiene (WASH) sector, human psychology serves as the basis for models promoting positive and sustained behaviour change [11–14]. The Human-Centred Design process, which includes the participation of community members in the process of co-designing a schistosomiasis behaviour change intervention, was applied in Tanzania [15]. Community involvement in designing WASH interventions for NTD control was piloted in Tanzania and resulted in increased awareness and some behavioural changes [16]. Intense health education and training of local volunteers to educate others has effected meaningful change in Ghana [17]. However, even a targeted health education intervention may not guarantee the desired outcome for elimination, as seen in China, due to inadequate stakeholder engagement [18]. Some other behaviour interventions have yielded similar mixed outcomes [19–23].

The 2021–2030 WHO NTD Road Map [4] for elimination emphasises accelerating programmatic action, intensifying cross-cutting approaches and changing operating models and culture to facilitate country and local ownership of NTD programmes. The move toward achieving this goal will require proxy measures outside of MDA. The focus for behaviour change should therefore not only be on the individual but rather on the whole community. This is because the community structure and norms can affect individual health behaviours, which are at the core of achieving success in eliminating disease [24]. Furthermore, a combination of control methods is required for complete elimination of schistosomiasis, as in the case of Japan [8]. In combining behaviour change approaches for WASH and schistosomiasis, the added complexity of the risk of water contact behaviour means any intervention should carefully consider the contextual, environmental and psychosocial factors.

The aim of this study was to explore immersive theatre in combination with community stakeholder awareness building as a behaviour change strategy in schistosomiasis control. We adapted the Risk, Attitude, Norms, Ability and Self-regulation (RANAS) framework [13, 25] because it fitted the factors which will affect WASH and water contact practices. The RANAS approach to systematic behaviour change involves four phases: (i) to identify potential behavioural factors; (ii) to measure the behavioural factors identified and determine those steering the behaviour; (iii) select corresponding behaviour change techniques (BCTs) and develop appropriate behaviour change strategies; and (iv) implement and evaluate the behaviour change strategies. We incorporated role play, specifically using the Acting for Health (AfH) theatre-based technique as a way of highlighting these contextual, environmental and psychosocial behavioural factors by tapping into the concept of storytelling within community nuances. This was in combination with cohort awareness raising and capacity training over a 5-day period. Storytelling has been widely used for imbibing moral lessons in many African cultures. To our knowledge, a theatre-based BCT intervention with such an extensive and immersive stakeholder face-to-face contact duration has not been previously applied in the context of a schistosomiasis-WASH behaviour change assessment.

## Methods

### Location

Four rural communities in Tanzania and one semi-urban town in Ethiopia were chosen as study locations. The four rural communities, Kigongo, Mwakalima, Chole and Nyangholongo, are located along the Lake Victoria basin of Misungwi District, Mwanza region, northwestern Tanzania. The one semi-urban town, Kemise, is the administrative centre of Oromia zone of Amhara region, Ethiopia. Kigongo is a village situated on Lake Victoria and is a major ferry port; Chole is a fishing village also on Lake Victoria; and the villages of Mwakalima and Nyangholongo are not situated directly on Lake Victoria but are close to scattered ponds and paddy farming activities. Schistosomiasis is not only prevalent in Lake Victoria but also in paddy farms and many ponds in the Mwanza region. The Ethiopian town of Kemise has a river that flows through it, which is a source of human water contact.

### Study design

**Fig. 1:**
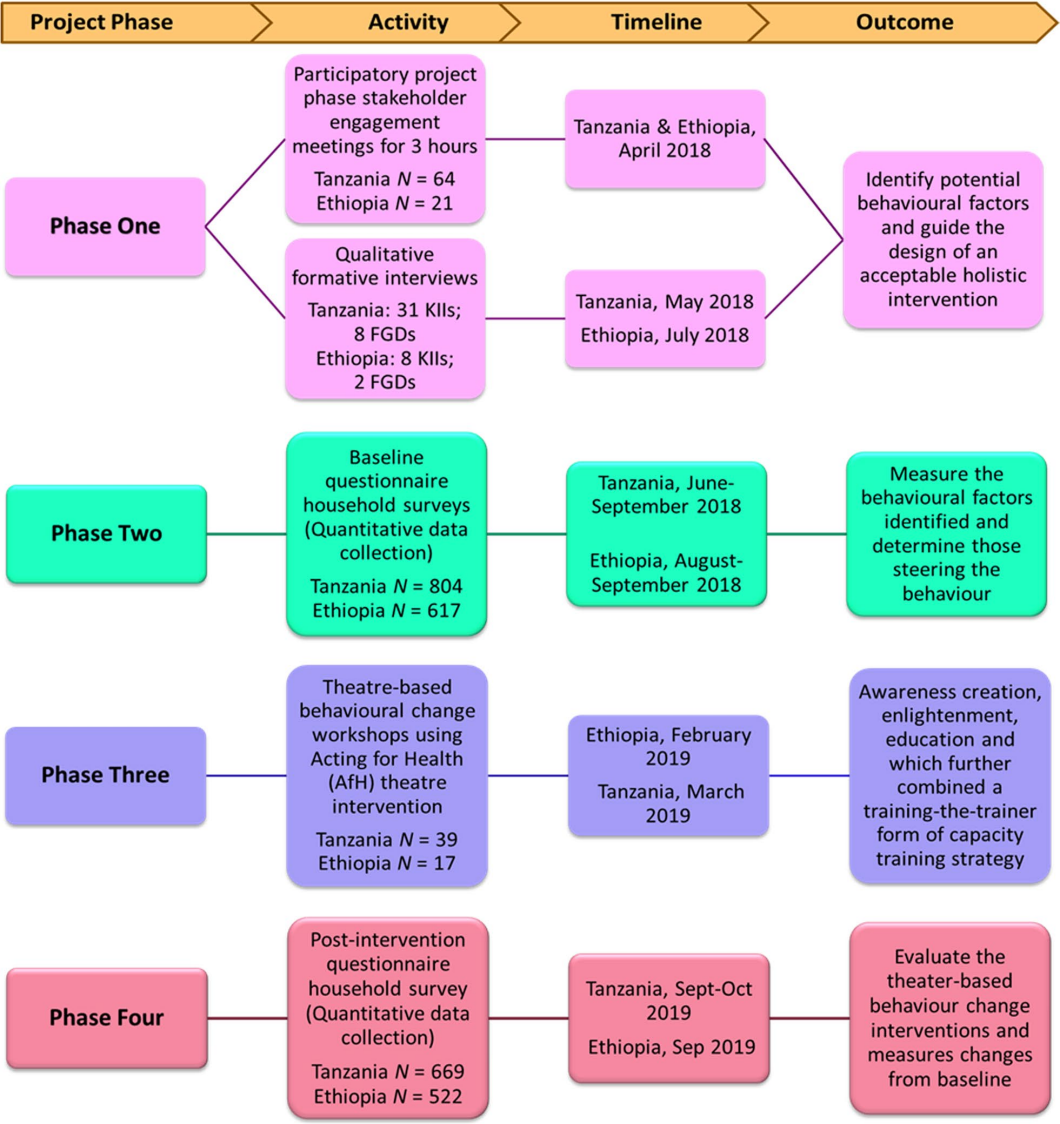
Flow diagram of the project phases using the RANAS approach to systematic behaviour change. Abbreviations: FGDs, Focus group discussions; KIIs, Key informant interviews; *N*, number of participants; RANAS, Risk, Attitude, Norms, Ability and Self-regulation

The four phases of the RANAS framework that were adapted for this study (Fig. 1) using a theatre-based behaviour change intervention and mixed-methods approach are described in detail in the following sections.

#### Phase one

Participatory project stakeholder engagement meetings were held, and outcome notes were taken to interpret findings from the sessions. Qualitative formative interviews were then carried out. Eight focus group discussions (FGDs) and 31 in-depth and key informant interviews were conducted in the four communities in Tanzania. Eight key informant interviews and two FGDs were conducted in the town in Ethiopia. Respondents included representative community members, local authority workers, regional and national governments, and representatives of occupational workers. A senior researcher or research assistant led the discussions using a simple topic guide (Additional file 1: Text S1), while a note taker managed audio recordings and took written notes of the meetings. Findings from the community meetings and emerging themes identified from the qualitative interviews were used to design the quantitative questionnaire surveys.

#### Phase two

Phase two comprised baseline (BL) questionnaire household surveys. In order to have a 95% level of confidence that the sampling for communities were within ± 5% of the true value for a given region, a sample size was determined after adjusting for the cluster sampling design and the assumed 97.5% response rate. The size was calculated for each group of adult men and women according to the demography. At BL, 804 questionnaires were administered in Tanzania (201 in each village) and 617 in Ethiopia. Questionnaires were translated into Kiswahili (Tanzania) and Amharic (Ethiopia). The survey questions and responses provided BL understanding of the knowledge, attitude, practices and perceptions of schistosomiasis, treatment-seeking behaviour, WASH and barriers to adopting adequate control measures.

#### Phase three

Behavioural change workshops using AfH theatre interventions were implemented. The AfH methodology and toolsets (detailed in Additional file 1: Text S2) uses creative theatre techniques for role play, the basis of which is an active listening paradigm used to explore issues and then to draw out solutions from the workshop participants. It gives a voice to all community groups by pulling from the lived experience of participants, using the different issues and perceptions they have around water contact and schistosomiasis, as well as their sense of personal responsibility, duty and agency to affect their own lives.

In Tanzania, two AfH workshops were held, in Mwakalima and Kigongo, respectively, and one workshop was held in Kemise, Ethiopia. The cohort of workshop participants included community representatives, occupationally exposed people (e.g. fishermen, paddy farmers and horse cart men), community health workers (CHWs), local authority workers, regional and national government representatives, mothers from the village, students and children (11–17 years old) (Additional file 1: Table S1). The theatre intervention was based around five daily sessions, each lasting 3 h, from 9 am to 12 noon every morning for 5 days, with 16–20 workshop participants for each location. There were 56 participants involved in all three workshops.

Collaborating researchers involved in the study worked together with the AfH team to ensure the right messaging was conveyed, and translators were used to communicate this in Kiswahili (Tanzania) and Amharic (Ethiopia). Prior to the theatre interventions, preliminary findings of the BL survey on behavioural factors revealed some awareness of symptoms of schistosomiasis. However, the survey also revealed gaps in understanding of the transmission routes, snail hosts, life-cycle of schistosomiasis and control measures and, consequently, the need for increased awareness and education of these topics. This informed part of the strategy for the education during the theatre intervention workshops and role play rehearsals.

At the end of the workshops, the play was performed to the community audience, and was filmed. In Tanzania, the film was shown in the other two communities, Chole (the viewing centre) and Nyangholongo (open air screening). The film was used as an awareness creation intervention in these two communities and as a means of measuring the impact of watching a local video production compared with watching a live dramatisation sketch by known community members or familiar faces. Likewise, the potential cost savings on disseminating films when compared to live performances. The film could also serve as an advocacy tool by stakeholders. Over 750 people, including children, watched the final performances of the play in the three locations, with 122 people, including children, watching the film in Chole (37) and Nyangholongo (85). Selected images from the intervention workshops are shown in Additional file 2: Figure S1. A sample of scenes from a complete drama sketch is shown in Additional file 1: Text S2 along with links to video recordings of the workshop sessions and films. The cohort of workshop participants who were local to the community made individual plans for peer and communal dissemination of the information learned at the workshops, thereby serving as community champions (Additional file 1: Table S1).

#### Phase four

Phase four consisted of post-intervention (PI) questionnaire household surveys. A gap of 6 months was allowed between workshop implementation and the PI survey to give the cohort participants enough time for informal community health and peer education. This time period was considered to be sufficient to give an indication of any intervention impacts. It also reduced the likelihood of data collection being associated with the intervention in the minds of participants and the associated risk of reactivity or respondent bias. In Tanzania, there was a participant return rate at the post survey of 83% (669/804 participants), of whom 65% (522/804) were successfully matched to the BL demographic data and ID numbers. In Ethiopia, the local security situation hindered the ability of data collectors to reach some participants during the PI survey. However, there was still a participant return rate of 70% (430/617 participants), of whom 369 (60%) were successfully matched to the BL data.

### Community grouping design

To effectively assess any changes from BL attributed to the interventions, community respondents at the post survey were divided into four groups in Tanzania. Given the nature of a large community setting where people were going about different daily activities, and where live plays and films were to be aired in public areas to the whole village, it was difficult at the planning stage to fit respondents into groups. Therefore, there was no enforcement on who should belong to which group. Rather, the groups were allowed to form organically. The questions used to indicate who belonged to which intervention group were included at the end of the questionnaire to reduce the level of bias which could be attributed by being asked those questions at the start. The choice of measure for behavioural outcomes requires careful attention as there may be many reasons why self-reporting is not a valid measure of actual behaviour [26]. To ensure we were not asking leading questions and to avoid the participants telling us what they thought we wanted to hear, and not the actual truth, we confirmed and validated survey responses of reporting positive change by using an open-ended question to ask how behaviour had actually changed. This further allowed for careful appraisal of whether the expected cause-effect linkage occurred.

Due to security challenges in Kemise, Ethiopia which started during the period of the research, detailed follow-up plans involving the select community workshop cohort could not take place and, consequently, the grouping was not applied. However, the workshop cohort from the Health Centre made plans to deliberately include lessons learnt around schistosomiasis knowledge and transmission into their general health awareness delivery plan.

### Data analysis

Audio recordings from qualitative interviews were transcribed and translated into English. Transcripts were entered as a Word document to facilitate text searching, coding and analysis. Three researchers used a word-by-word analysis to identify and categorise explanations and classify the overarching themes from transcripts into memos [27]. A combination of preset themes identified from the findings of the participatory project phase meetings and emergent categories from the qualitative data were used. Two of the researchers reviewed the memos and developed a coding framework using an iterative process. One researcher coded the memos to ensure relevant themes were discussed and included in the BL questionnaire and in the intervention workshops.

Questionnaire responses were entered into Excel (Microsoft Corp., Redmond, WA, USA) spreadsheets and translated into English. Quantitative data analysis was carried out using the statistical software package StataIC 16.1 (StataCorp LLC, College Station, TX, USA). As there was a mix of WASH- and schistosomiasis-related water contact behaviours, we applied standard percentage change and paired t-tests to evaluate changes from BL to PI survey, instead of the regular RANAS doers and non-doers evaluation. This modification was more suited to the schistosomiasis and wider NTD sector, and more practical for the large number of participants involved. We applied the Pearson Chi-square test to determine the effect of gender, age and educational level on the results for Ethiopia and Tanzania. The Pearson Chi-square test was also used to assess the impact of participating in the different intervention groups in Tanzania.

## Results

### Participatory project phase and qualitative formative findings

During the participatory sessions, important themes on risk awareness, attitude, perceptions and understanding began to emerge within the context of their lived experiences. These included problems associated with schistosomiasis misconceptions and stigma; religious and traditional reasons for bathing in contaminated rivers; distance to accessing health services; schistosomiasis being believed to be part of the male adolescent growth phase; using alternative traditional medicine; among others. There was a general awareness of the symptoms of schistosomiasis. However, in both Tanzania and Ethiopia there was a gap in participants’ understanding of the transmission routes, life-cycle of schistosomiasis and control measures.

The qualitative KII and FGD findings further revealed that many participants were aware of the existence of schistosomiasis in their communities and correctly pointed out some schistosomiasis symptoms, such as passing blood in urine, in the case of *Schistosoma haematobium* infection. Generally, the disease was viewed as a routine part of their life and a cause for poor health in children. Many interviewees also complained about lacking clean water sources in their local settings, not necessarily because of schistosomiasis, but in terms of other waterborne diseases, including cholera, diarrhoea and amoebic dysentery. Again, there were gaps in participant’s awareness and understanding of the disease transmission routes, the life-cycle, the preventive and control measures associated with sanitation and risky water contact behaviours with contaminated water bodies. Examples of responses and narrative quotations are shown in Table 1 (more quotations are given in Additional file 1: Table S2).

**Table 1:** Emergent themes and narrative quotations from focus group discussions used to explore schistosomiasis knowledge, attitude and perception of risks, transmission and control measures

Themes	Narrative quotations
Attitude and perceptions on risks and severity	*Female FGD*: “The most common health problem which disturbs our community is bilharzia, children several times pass blood during urination.”; “We can rank malaria, there is typhoid and UTI and bilharzia disease followed.” *Male FGD*: “I suffered from this disease when I was in standard one until I reached standard five. I was suffering from stomach pain and discharging blood in the urine, also pain during urination.”; “I was very sick but by the time when I was 15 years the disease stopped, and I was not urinating blood anymore.”; “I know at the time I was at school we were taught that water from the rivers is safe because they are flowing water because they flow with the bacteria in it. We were taught that any flowing water is safe.”
Knowledge on transmission	*Female FGD*: “For us women, when we urinate, we use to squat so when an infected person has urinated and another goes to squat and urinate there, she may acquire bilharzia because the bacteria have steam which enables them to come up to you and can be transmitted that way.”; “Bilharzias can be caused by sexual intercourse. If one partner has it, he/she can transmit to the other.” *Male FGD*: “In the past schistosomiasis was spreading through clans.”; “If I have schistosomiasis and I have a wife, when I give birth to children, they will surely have it, that's how it was. When you give birth to the children, after they have grown up they will start urinating blood because I had it. If the father has it then even the children must have.”; “You would find someone is eating food with too much salt, then you would find he is acquiring that disease.”
Treatment-seeking behaviour	*Female FGD*: “Many people don’t know that bilharzia can be treated in the hospital so they use traditional medicines.”; “In the programme of distributing medicines at schools, it is until the parents are educated about these medicines, because some of the parents refuse and complain that the children will die. If they will be educated, the system will be continuous.” *Male FGD*: “Some children who have been given tablets without being tested and maybe had allergy and the drug severely affected them, while others are losing consciousness, others get this rashes and this causes parents to prevent their children to go to school when they hear that pupils will be given tablets.”; “Adults are not included in free drugs distribution.”
Control	*Female FGD*: “There isn’t any way for preventing bilharzia so that you won't acquire it again, only the medicine will help prevent that disease. I don't see any other way simply because if we could have done something before then we wouldn't be suffering from bilharzia.” *Male KII:* “I think that the best way is toilets. This way will also protect us from so many things, and it will be treatment for curing many diseases affecting the stomach. For that reason, I support this method, in fact it would be the first of all from other methods.”; “In order to eliminate schistosomiasis, government must construct water infrastructure to reduce the number of people who are going to the lake.”
Self-regulation and responsibility	*Male FGD*: “For the side of farmers, I am asking government to think and look to minimise the price of those equipment which protect people from acquiring bilharzia. For example, as I am speaking if you come to Moshi and enquire. I should wear long gloves and other things. I went to the agriculture inputs shop and found those waterproof gumboots which their size reach here and are tight, and that raincoat which is made of plastic to prevent water penetration, and they told me total price is 360,000 shillings. I have five youths, including me we are six, how can I afford that cost to protect ourselves from bilharzia.” *Female FGD*: “I can contribute 5000 shillings per month for water service.”; “According to my financial capacity I can contribute 3000 shillings per month.”

*FGDs* Focus group discussions,* UTI* urinary tract infection

### Play versus film audience survey

The audience who watched both the play and film for the same drama sketch, learnt several lessons. However, the themes mentioned by the interviewees differed in a few categories, for example on water contact, as shown in Table 2. This might be attributed to the roles of some characters being played by community members, which shifts the attention more to particular themes.

**Table 2:** Interview answers according to watching the play versus the film per category

Play or film? (number of interviewees)	Mentioned themes										
	Urination location	Government to bring clinic/water	Go to hospital	Water contact	Effects of schistosomiasis	Sanitation/clean environment	Use clean water	Symptoms	Educate others	Transmission	Learnt something incorrect
Play (39)	54% (21)	21% (8)	21% (8)	31% (12)	8% (3)	36% (12)	38% (15)	21% (8)	21% (8)	18% (7)	5% (2)
Film (20)	45% (9)	10% (2)	30% (6)	80% (16)	10% (2)	30% (6)	35% (7)	20% (4)	10% (2)	20% (4)	10% (2)

Values in table are given as the percentage (number) of people who mentioned it for each category

### Quantitative BL versus PI questionnaire survey

The demographic characteristics of participants at BL and PI are shown in Table 3. The information captured gender, age and educational level of participants.

**Table 3:** Demographic characteristics of survey respondents in Ethiopia and Tanzania

Demographic characteristics	Ethiopia		Tanzania	
	Baseline, *N* = 617 *n* [%]	Post intervention, *N* = 426 *n* [%]	Baseline, *N* = 804 *n* [%]	Post intervention, *N* = 669 *n* [%]
*Gender*				
Male	329 [53]	224 [53]	379 [47]	322 [48]
Female	288 [47]	202 [47]	425 [53]	347 [52]
*Age*, years				
15–24	139 [23]	43 [10]	83 [10]	77 [12]
25–34	231 [37]	112 [27]	229 [29]	137 [20]
35–44	116 [19]	118 [28]	210 [26]	146 [22]
45–54	80 [13]	86 [20]	220 [27]	177 [26]
55+	51 [8]	61 [14]	62 [8]	132 [20]
No response recorded	–	6 [1]	–	–
*Educational level*^a^				
Unable to read & write	161 [26]	129 [30]	140 [17]	140 [21]
Only able to read and write	47 [8]	59 [14]	–	–
1st cycle primary (grade 1–4)	49 [8]	30 [7]	108 [13]	87 [13]
2nd cycle primary (grade 5–7)	164 [26]	77 [18]	461 [58]	368 [55]
Secondary education (grade 1–6)	128 [21]	50 [12]	87 [11]	69 [10]
Preparatory education	24 [4]	30 [7%	–	–
University degree	44 [7]	21 [5]	8 [1]	5 [1]
No response recorded	-	30 [7]	–	–

Values in table are given as the number (*n*) with the percentage in square brackets

^a^Education variables for both countries are based on the accepted national standards

Complete results of the BL and PI surveys are given in Additional file 1: Table S3 (Tanzania) and Additional file 1: Table S4 (Ethiopia) and are summarised below using the adapted RANAS framework factors.

#### Risk factors (Knowledge and awareness of the danger of schistosomiasis)

Table 4 shows results by respondents in Tanzania and Ethiopia on understanding the risks, severity and danger of schistosomiasis. In Tanzania, at PI, there was an increased awareness of the danger of schistosomiasis by the male respondents compared to the female respondents (Additional file 1: Table S3). However, the female respondents had a higher baseline awareness of the risk factors for the disease. On the other hand, in Ethiopia (Additional file 1: Table S4), the baseline awareness of male respondents was higher compared with female respondents. Also, the younger the respondent the less dangerous they ranked schistosomiasis.

**Table 4:** Summary of questionnaire survey results at baseline and post intervention for risks and treatment seeking

Questionnaire survey questions	Tanzania (*N* = 522)			Ethiopia (*N* = 369)		
	Yes, as selected option^a^ BL [%]	Yes, as selected option^b^ PI [%]	* P* value^c^	Yes, as selected option^a^ BL [%]	Yes, as selected option^b^ PI [%]	*P* value^c^
*Schistosomiasis affects both children & adults?*	88	95	0.0003	75	83	0.0048
*Do you think schistosomiasis is dangerous?*			0.0002			0.0219
Very dangerous	46	51		50	66	
Dangerous	43	45		44	27	
Slightly dangerous	9	4		4	3	
Not dangerous	2	0		2	4	
*Where is the right place to seek treatment?*			0.0006			0.0195
Health facility/centre/hospital	95	99		95	98	
Drug shop	3	1		4	2	
Traditional healer	1	0		1	0	
Other	1	0		0	0	
*Have you or your child ever been treated for schistosomiasis before*?	49	61	0.0000	27	–	–

^a^Values in table are given as the percentage of baseline respondents

^b^Values are given as percentage of post-intervention survey respondents

^c^Paired t-test,* P* value

#### Attitude factors (Treatment-seeking behaviour)

Table 4 shows that there was an increase, in Tanzania, of respondents—or their children—who had been treated for schistosomiasis at PI. The significant increase was linked to a shift at both the gender (*P* = 0.0001) and educational levels (*P* = 0.003) (Additional file 1: Table S3). Male respondents were more likely than female respondents to seek hospital treatment, with the former who had accepted medication increasing from 56% to 73% at PI, while female respondents who had accepted medication increased from 43 to 50%. Those who had achieved primary educational level and above showed an increased acceptance of medication PI, while acceptance did not increase among those with no education at all. In Ethiopia, the older the respondent the more likely they would seek treatment in a drug store instead of a medical centre.

#### Norm and contextual factors (Community control and prevention of schistosomiasis)

**Fig. 2:**
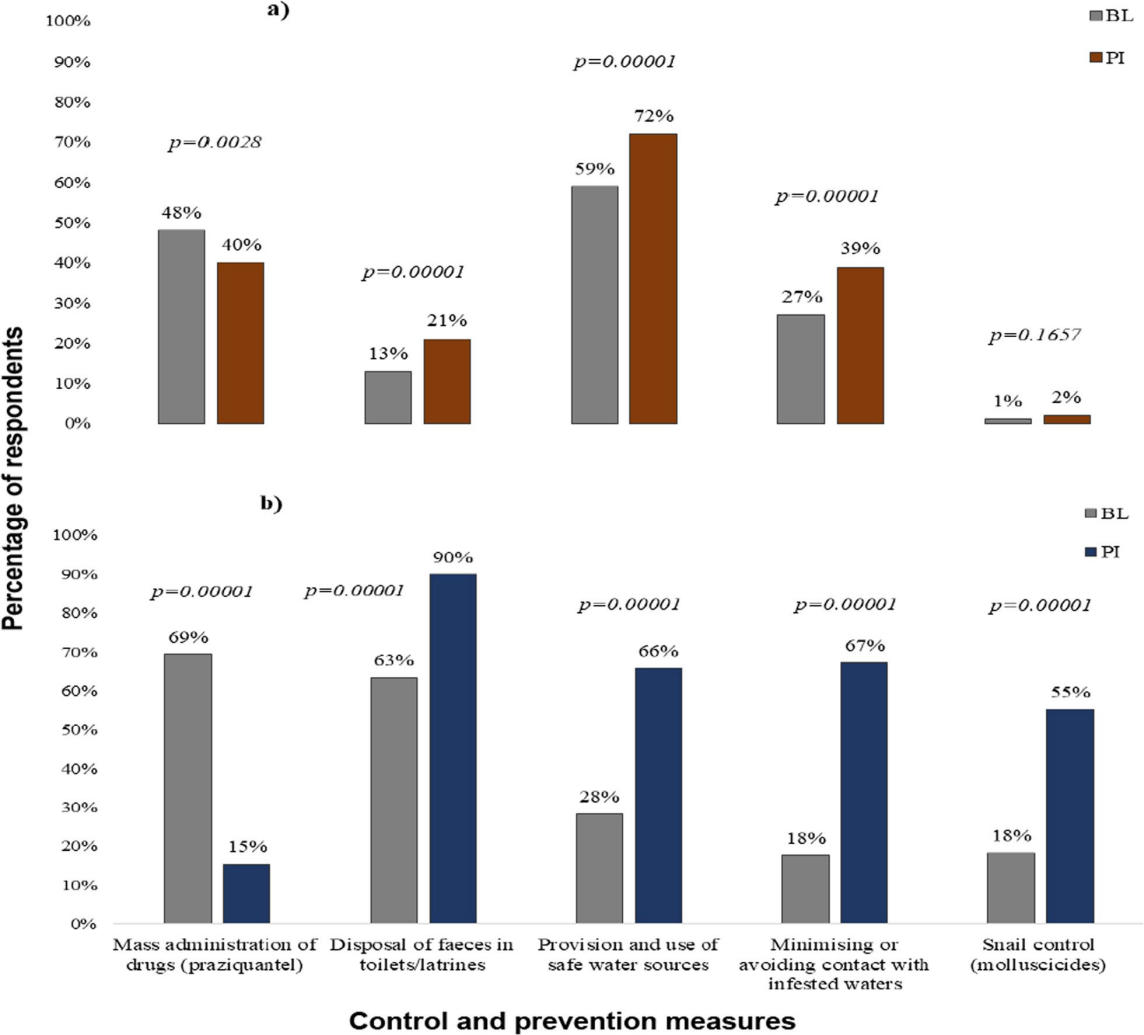
Responses on schistosomiasis control and prevention measures at BL and PI and percentage of respondents (*y*-axis). **a** Tanzania, **b** Ethiopia. Abbreviations: BL, Baseline; PI, post-intervention survey

Selected prevention or control measures at BI and PI are shown in Fig. 2. In Tanzania (Fig. 2a; Additional file 1: Table S3), educational level had a significant effect on responses. The lower the educational level, the less likely the respondent was to suggest the provision of safe water sources (BL: *P* = 0.011; PI: *P* = 0.0001); a similar response was seen for minimising water contact (BL: *P* = 0.0001; PI: *P* = 0.0001). Regarding MDA, those with no education and those with the first-cycle primary level rated it higher than those with second-cycle primary level and above (*P* = 0.0001). Critically, the realisation of transmission being linked to sanitation resulted in more respondents taking responsibility and paying for building their latrines (BL: 10%, PI: 41%). In Ethiopia, at baseline, MDA was considered the preferable option due to the dominance of MDA campaigns to date (Fig. 2b). PI, there was a notable switch to safe water, minimising water contact and disposal of urine/faeces in toilets.

#### Ability and contextual factors (Water contact behaviours)

The main reason respondents used infested water sources for domestic, recreational and occupational needs in Tanzania (Additional file 1: Table S3) was because these were the only available sources (BL: 35%; PI: 67%). Respondents in Tanzania (BL: 94% and PI: 94%) and Ethiopia (BL: 99%, PI: 94%) were willing to avoid/minimise all existing contact with contaminated water sources if they had an alternative clean source. In Tanzania, age or gender did not influence choices, but educational level had some influence PI (*P* = 0.018), with those having a primary-level education and higher (94–100%) saying yes compared to those not educated at all (86%).

#### Self-regulation and contextual factors (Responsibility)

As shown in Additional file 1: Tables S3 and S4, the majority of respondents in both Ethiopia and Tanzania supported their respective government overseeing the installation of water infrastructure for the control of schistosomiasis (Ethiopia, BL: 67%, PI: 76%; Tanzania, BL: 71, PI: 72%). In Ethiopia, the intervention increased a belief in the potential and need for community-led initiatives in maintaining installed water infrastructure (BL: 41%, PI: 59%). At the same time, the percentage of those who opted for government to maintain water infrastructure decreased (BL: 47%, PI: 18%). This switch was primarily driven by male respondents. The opposite trend was observed in Tanzania, where PI, there were more respondents who supported the government being the sole entity responsible for the maintenance of water infrastructure (BL: 13%; PI: 57%) and to a lesser extend individual users (BL: 4%; PI: 19%).

#### Effect of intervention groupings on overall attitude and behaviour (Tanzania only)

In Tanzania, the composition of the intervention groups is shown in Table 5. The intervention groupings were allowed to form organically which led to groups with different sizes. However, this did not affect the comparability of results between those respondents who participated in one or more forms of the intervention (groups 1–3) and those who did not have any form of intervention (group 4, serving as the control group).

**Table 5:** Breakdown of intervention groups in Tanzania

Groupings^a^	Description	Village/Number of respondents at post intervention				
		Kigongo	Mwakalima	Chole	Nyangholongo	Total
Group 1	Those who viewed the play (drama/film) and have had some follow-up discussions with either the community health workers, other workshop cohort or through attending a community group meeting	44	51	5	4	104
Group 2	Those who viewed the play but have not had any follow-up discussions	0	5	2	6	13
Group 3	Those that did not view the play but have had some follow-up discussions on schistosomiasis with either the community health workers, other workshop cohort or through attending a community group meeting	72	43	20	35	170
Group 4 (control)	Those that did not view the play and have not had any follow up discussions (which served as control group)	50	84	132	116	382
Total		166	183	159	161	669

^a^Groups 1–3 included respondents who participated in one or more forms of the intervention; group 4 included respondents who did not have any form of intervention (control group)

At PI, a respondent’s participation in one or more forms of the intervention (groups 1–3) had a higher positive impact compared to those who did not have any form of intervention (group 4), as shown in Additional file 1: Table S3. Those who participated in groups 1–3 were more likely to mention water contact as a mode of transmission (96–100%) compared to those in group 4 (91%) (*P* = 0.023). Participants in groups 1–3 were more likely to seek hospital treatment and accept medication (61–76%) compared to those in group 4 (58%), although the difference was not significant (*P* = 0.167). Safe water was mentioned by 78–85% of those in groups 1–3 and by 68% of those in group 4 (*P* = 0.037). Similarly, those in groups 1–3 were more likely to mention sanitation compared to those in group 4 (*p* = 0.044). Furthermore, those in groups 1–3 were more likely to say their behaviour, attitude or understanding of schistosomiasis, transmission and prevention had changed (83–92%) compared to those in group 4 (61%) (*P* = 0.0004), as shown in Table 6.

**Table 6:** Summary of results on reported changed behaviour

	Group 1	Group 2	Group 3	Group 4	Total
*Has your behaviour and attitude changed as a result of this interventions? Response = Yes (P value = 0.0004)*	84% (71)	92% (12)	83% (136)	61% (30)	80% (249)
*How has your behaviour changed? (Taken from open-ended responses)*					
I have built a latrine and use it and I no longer openly defecate or urinate	38% (27)	58% (7)	30% (41)	27% (8)	33% (83)
I now use clean water from closed well, tube well, rainwater harvesting	56% (40)	42% (5)	49% (67)	60% (18)	52% (130)
I am no longer bathing or swimming in lake or pond	14% (10)	25% (3)	15% (20)	30% (9)	17% (42)
I have stopped my children from playing or swimming in lake or pond	11% (8)	8% (1)	9% (12)	17% (5)	10% (26)
I now take medication	6% (4)	0 (0)	4% (5)	0 (0)	4% (9)
I have learnt much^a^	17% (12)	8% (1)	24% (32)	20% (6)	20% (51)
I use gumboots	1% (1)	8% (1)	3% (4)	3% (1)	11% (7)
*Has your behaviour and attitude changed as a result of this interventions? Response = No*	16% (14)	8% (1)	17% (28)	39% (19)	20% (62)
*Any reason why your behaviour has not changed? (From open-ended responses)*					
I have not changed because we still have the same contaminated water source	79% (11)	100% (1)	89% (25)	26% (5)	68% (42)
I have not changed because of my work	0 (0)	0 (0)	11% (3)	0 (0)	5% (3)

Values in table are given as the percentage of respondents, with the number in parentheses

^a^Nothing specific was mentioned and, therefore, no change was assumed

Age, gender and educational level did not have any significant effect on the responses of the groups. However, it should be noted that the 61% in group 4 represents 30 out of the 49 respondents who responded to the question, from among the total of 382 participants in the group. The 249 participants in groups 1–4 who answered yes to changing behaviours were further asked to explain the one or two ways in which their behaviour had changed; of those asked, 80% provided open-ended responses to back their claim (Table 6). Interestingly, 20% mentioned they had learnt much without saying anything specific, and we can possibly assume they had not actually changed their behaviour, or they could not think of what to say when put on the spot. The evaluations in this respect therefore accounted for active psychological, social and physical environmental changes associated with the intervention [26].

## Discussion

We observed some differences between gender and educational levels in the study outcomes. For example, at the PI survey in the Mwanza (Tanzania) villages, males were more likely to seek hospital treatment, and the number of those who had accepted and taken medication had increased significantly compared to females. This observation is not unique to this study as similar outcomes have been observed in previous MDA programmes [28–30]. Our results indicate a direct relationship between educational level and awareness of control and prevention of schistosomiasis, topics which were discussed during the interventions. Overall, the intervention increased the awareness of schistosomiasis risks and addressed some of the issues needed to reduce its transmission. However, there is still the need for a concerted effort within the NTD community to improve understanding of the gender gap and to design interventions which specifically reach women as well as those with no education.

Effective stakeholder involvement of community groups in behaviour approaches can provide a sustained and resilient system [31]. Participatory learning by the selected cohort in Mwanza and their subsequent commitment to be community champions of the interventions ensured that the learnings and gains from the workshops were sustained and maintained amongst the participants. The planned educational strategy using peer learning was successful in the community. The participants reinforced and put into practice the knowledge gained during the intervention workshops, for example by distributing flyers (Additional file 2: Figure S2), forming school health clubs and raising awareness during village meetings. A high number of respondents from the intervention groups were recorded in the PI survey, and these respondents also showed a better understanding of the disease. Both outcomes are a testament to the voluntary work carried out by the cohort community champions and their commitment, as well as a legacy of our interventions, which were a first step in facilitating this community change. Similarly, in Kemise, many respondents had heard about the transmission of schistosomiasis from the health centre, another indication that the cohort participants from the community health centre had applied the inclusive teaching they had pledged during the intervention workshops.

Using community champions is one strategy for finding strengths and building capacities for improvement [32]. However, behaviour change can only be effective when all infrastructure for change is in place. Safe water provision was not an issue for Kemise respondents in terms of perceived problems, or water being a barrier to control and eradication. This was most likely because the majority of this semi-urban town (approx. 80%) already have pipe-borne water. On the other hand, safe water was a huge barrier for the rural Mwanza villages. These communities had no water infrastructure in place at the time of the survey and this situation has not changed; consequently, we cannot determine some of the changes in behaviour that could or would have taken place. Nevertheless, the positivity in making some informed changes by the community demonstrated an intention and willingness to change [13]. For example, the numerous observed and reported numbers of household latrines recently built, the increased uptake of preventive chemotherapy and the willingness to learn about the transmission and prevention of schistosomiasis, are all positive steps.

During the intervention, we were mindful of socio-cultural, religious, economic and/or historical reasons for having contact with contaminated river, lake or pond water, and we consciously avoided any blame game. In Kemise for example, the spiritual cleansing with river water during the thirteenth month of “Pagume” could have influenced why some respondents would still not avoid infested waters, especially since the PI questionnaires were completed near the time of the celebrations. Therefore, for behaviour to change, people’s mindsets must first change [25]. When knowledge is activated in the mind, beliefs and emotions rise to the fore, and an intention to perform a particular behaviour emerges, eventually resulting in observable behaviour [25]. Individual behaviour can also be encouraged or constrained by the community norms and behaviour of families, social groups, communities, organisations and policy-makers [24]. For the global NTD-WASH sector to achieve the desired behaviour changes, a total systems approach and multisectoral coordination are needed. These initiatives will involve a country’s national, local and community leaders in order to be able to tackle all socio-economic and other barriers to change, as shown in the WHO Road Map [4].

The intervention contributed to a large extent in changing awareness and attitudes toward infrastructural and behavioural actions which serve as preventive measures for schistosomiasis. In the case study villages in Mwanza, there was a shift toward holding the government responsible for the provision of water infrastructure. In this respect, our study has raised the awareness of citizens and mobilised them to recognise their basic human right to free access to clean water from the state. Perhaps this is what is needed now for NTD-WASH and would set a precedent for transformative investments in safe water by low- and middle-income countries (LMIC) states toward using utility-scale service models [33]. Water provision for the poor should be seen as a service. For too long, it has often been treated as a product, resulting in many low-cost household water treatment measures, if used at all or even if used correctly, still failing to reach measurable human health benefits [33–35]. Moreover, schistosomiasis requires safe water for complete household domestic use and not just for drinking water. Providing centralised or decentralised state-subsidised utility-scale water service is expensive, but a price ultimately worth paying. Historically, universal access to water has only been possible with utility services [33], and this looks like the most suitable option for ensuring sustained water contact behaviour change toward eliminating schistosomiasis as a public health concern.

### Limitations

At the PI survey, the return rate for responses could have been better and would have been improved with more rigour in ascertaining the BI participants. The use of ethical biometric data capture could be one way forward for BI and PI or endline survey studies.

There were a few misunderstandings in knowledge which cut across all participants regardless of whether they participated in an intervention or not. For example, many participants mentioned water contact as well as drinking dirty water causing schistosomiasis, probably because they were still informed not to drink dirty water. Nevertheless, the knowledge that skin contact causes the disease had increased. Again, we assumed the notable switch on selecting preventive chemotherapy as a control measure reflected participants’ understanding of the importance of other integrated control measures, but this may not be so. The importance of MDA in decreasing morbidity and excreted eggs should always be emphasised as a benefit to decrease transmission. There is still scope for fine-tuning interventions to ensure delivery of the right knowledge messaging and ensuring this filters down correctly. The interventions are best viewed as an iterative process, with each iteration drilling down further into the root drivers of behaviour, and each one building on the previous.

A survey and observation of the community-based cohort participants at 16 weeks showed that 90% were still involved in disseminating what they had learned at the workshops. These community champions had faced a few challenges in educating peers, especially in terms of having the right answers to posed questions. This could have been avoided with a better planned follow-up training process and printed materials in place.

## Conclusions

The AfH theatre BCT and capacity training intervention workshops described in the present study were successful in building relationships with the local community and for engagement with people in Tanzania and Ethiopia. This study demonstrated that a theatre-based BCT can be an effective tool for communicating sustainable behavioural change messages, creating dialogue, bridging the gap and untangling the issues of mistrust and misconceptions. Participants were able to rethink problems from a different viewpoint in an interactive, immersive and non-prescriptive way, and were given a voice to share their lived experiences. There was a significant increase in the awareness of the transmission of schistosomiasis amongst participants, leading to positive community changes and commitments. With appropriate sensitisation, mobilisation, education and engagement approaches, people were more open to minimising risk-associated contact with contaminated water sources, using alternative, improved water supplies and practicing adequate sanitation and hygiene and, thereby, creating lasting behaviour change needed to ensure long-term sustainability. Our study contributes to the existing body of literature on behaviour change interventions in sustaining positive health outcomes. Our recommendation is for all NTD interventions using preventive chemotherapy and/or WASH to include raising awareness and knowledge around transmission, prevention and long-term morbidity. The COVID-19 pandemic has highlighted the importance of individuals understanding how to minimise risk of exposure, just as much so understanding the symptoms and medical treatments available post-infection; and the same is certainly true for schistosomiasis.

## Supplementary Information

**Additional file 1: Text S1**. Qualitative interviews and focus group discussions topic guide and questions. **Text S2**. Acting for Health methodology. **Table S1**. Intervention workshop cohort and drama and film audience survey responses. **Table S2**. Emergent themes and narrative quotations from formative qualitative findings. **Table S3**. Quantitative questionnaire survey results for baseline and post intervention for Tanzania. **Table S4**. Quantitative questionnaire survey results for baseline and post intervention for Ethiopia.

**Additional file 2: Figure S1**. Some selected images from intervention workshops. **Figure S2**. Flyer with life cycle, transmission and control in Tanzania

## Data Availability

The authors confirm that all data underlying the findings are fully available within ethical considerations. All relevant data are within the paper and its supplementary information files.

## Abbreviations

AfH: Acting for health
BCC: Behavioural change communication
BCT: Behaviour change technique
BL: Baseline
CHWs: Community health workers
FGDs: Focus group discussions
KIIs: Key informant interviews
LMIC: Low- and middle-income countries
MDA: Mass drug administration
NTDs: Neglected tropical diseases
PI: Post intervention
RANAS: Risk, Attitude, Norms, Ability and Self-regulation
WASH: Water, sanitation and hygiene
