# Supplementary material for: A novel theatre-based behaviour change approach for influencing community uptake of schistosomiasis control measures
Source: Parasit Vectors. 2022 Aug 25;15:301. doi: 10.1186/s13071-022-05421-5 (PMC9406251; doi:10.1186/s13071-022-05421-5)
Supplement: Supplementary file 1 — Additional file 1: Text S1. Qualitative interviews and focus group discussions topic guide and questions. Text S2. Acting for Health methodology. Table S1. Intervention workshop cohort and drama and film audience survey responses. Table S2. Emergent themes and narrative quotations from formative qualitative findings. Table S3. Quantitative questionnaire survey results for baseline and post intervention for Tanzania. Table S4. Quantitative questionnaire survey results for baseline and post intervention for Ethiopia. [file 13071_2022_5421_MOESM1_ESM.zip › Table S1.docx]

### TABLE S1: COHORT (COMMUNITY REPRESENTATIVES ONLY) RESPONSES FROM DAY 1 AND DAY 5 DURING INTERVENTION WORKSHOPS AND PLAY/FILM AUDIENCE RESPONSES IN TANZANIA

1. COHORT RESPONSES (EXCLUDING DISTRICT WASH AND NTD OFFICERS)

### Expectations of the intervention – Day 1

| **Key** | | |
| --- | --- | --- |
| 0% | 50% | 100% |

Table 1A: Participants when asked what they are expecting to learn, to do and to take away, by occupation. X% is the percentage of participants in that category giving that response. (#) is the number in that category giving the response.

| **Occupation** | **Wanted to learn how to prevent schistosomiasis** | **Hoping to educate others** |
| --- | --- | --- |
| Farmer (14) | 31% (4) | 31% (4) |
| Teacher (4) | 75% (3) | 75% (3) |
| Community Health Worker (4) | 50% (2) | 75% (3) |
| Village Officer (4) | 50% (2) | 50% (2) |
| Teenagers (4) | 75% (3) |  |
| Driver (3) | 67% (2) |  |
| Fishermen/sellers (3) Carpenter (1) | 75% (3)^1^ | 50% (2) |
| **TOTAL (37)** | 53% (19) | 39% (14) |

^1^3/3 fishermen said they wanted to learn how to prevent schistosomiasis, 0/1 carpenters mentioned this

Table 1B: Participants when asked what they are expecting to learn, to do and to take away, by village.

| **Village** | **Wanted to learn how to prevent schistosomiasis** | **Hoping to educate others** |
| --- | --- | --- |
| Kigongo (20) | 42% (8) | 37% (7) |
| Ng'Wakalima (17) | 65% (11) | 41% (7) |

Table 1C: Participants when asked what they are expecting to learn, to do and to take away, by level of education

| **Level of Education** | **Wanted to learn how to prevent schistosomiasis** | **Hoping to educate others** |
| --- | --- | --- |
| Below primary education (3) | 33% (1) | 33% (1) |
| Primary education (24) | 52% (12) | 22% (5) |
| Secondary education or above (10) | 60% (6) | 80% (8) |

Table 1D: Participants when asked what they are expecting to learn, to do and to take away, by gender

| **Gender** | **Wanted to learn how to prevent schistosomiasis** | **Hoping to educate others** |
| --- | --- | --- |
| Female (16) | 50% (8) | 50% (8) |
| Male (21) | 55% (11) | 30% (6) |

Table 1E: Participants when asked what they are expecting to learn, to do and to take away, by age category

| **Age Category** | **Wanted to learn how to prevent schistosomiasis** | **Hoping to educate others** |
| --- | --- | --- |
| <25 (6) | 83% (5) | 17% (1) |
| 25<X<40 (11) | 70% (7) | 40% (4) |
| 40<X<50 (11) | 45% (5) | 45% (5) |
| >50 (9) | 22% (2) | 44% (4) |

### Learning – Day 5

Table 2A: Results of what participants learnt, by occupation.

| **Occupation** | **Previously unaware of disease** | **Sanitation** | **Symptoms** | **Treatment of disease** | **Transmission /Prevention** | **Cycle of disease** | **Effects of disease e.g. cancer** | **Not surprised by anything** | **Form of communication** | **Solutions** |
| --- | --- | --- | --- | --- | --- | --- | --- | --- | --- | --- |
| Farmer  (14) | 23% (3) | 8% (1) | 15% (2) | 23% (3) | 64% (9) | 15% (2) | 31% (4) | 8%  (1) |  |  |
| Teacher  (4) |  | 25% (1) |  |  | 100% (4) | 75% (3) | 25% (1) |  | 25% (1) |  |
| Community Health Worker (4) |  | 50% (2) | 25% (1) | 25% (1) | 50% (2) |  |  |  |  | 25% (1) |
| Village Officer  (4) | 25% (1) | 50% (2) |  |  | 75% (3) |  |  |  | 25% (1) | 50% (2) |
| Teenagers  (4) |  |  | 25% (1) |  | 100% (4) | 75% (3) |  | 25% (1) |  |  |
| Driver  (3) |  | 67% (2) |  |  | 67% (2) |  |  | 33% (1) |  |  |
| Fish Seller/ Carpenter (4) |  | 25% (1) |  |  | 75% (3) |  | 25% (1) | 25% (1) | 25% (1) |  |
| **TOTAL (37)** | **11% (4)** | **25% (9)** | **11% (4)** | **11% (4)** | **73% (27)** | **22% (8)** | **17% (6)** | **11% (4)** | **8% (3)** | **8%**  **(3)** |

Table 2B: Results of what participants learnt, by village

| **Village** | **Previously unaware of disease** | **Sanitation** | **Symptoms** | **Treatment of disease** | **Transmission /Prevention** | **Cycle of disease** | **Effects of disease e.g. cancer** | **Not surprised by anything** | **Form of communication** | **Solutions** |
| --- | --- | --- | --- | --- | --- | --- | --- | --- | --- | --- |
| Kigongo  (20) | 11% (2) | 42% (8) |  | 11% (2) | 65% (13) | 16% (3) | 21% (4) | 11% (2) | 11% (2) | 11% (2) |
| Ng'Wakalima  (17) | 12% (2) | 6% (1) | 24% (4) | 12% (2) | 82% (14) | 29% (5) | 12% (2) | 12% (2) | 6% (1) | 6% (1) |

Table 2C: Results when asked what they learnt, what they took away and what surprised them, by level of education

| **Level of Education** | **Previously unaware of disease** | **Sanitation** | **Symptoms** | **Treatment of disease** | **Transmission /Prevention** | **Cycle of disease** | **Effects of disease e.g. cancer** | **Not surprised by anything** | **Form of communication** | **Solutions** |
| --- | --- | --- | --- | --- | --- | --- | --- | --- | --- | --- |
| Below primary education (3) | 67% (2) |  |  | 33% (1) | 67% (2) |  |  | 33% (1) |  |  |
| Primary education (24) | 9%  (2) | 30% (7) | 17% (4) | 9%  (2) | 67% (16) | 13% (3) | 13% (3) | 13% (3) |  | 4%  (1) |
| Secondary education or above (10) |  | 22% (2) |  | 11% (1) | 89% (8) | 44% (4) | 33% (3) |  | 33% (3) | 22% (2) |

Table 2D: Results when asked what they learnt, what they took away and what surprised them, by gender

| **Gender** | **Previously unaware of disease** | **Sanitation** | **Symptoms** | **Treatment of disease** | **Transmission /Prevention** | **Cycle of disease** | **Effects of disease e.g. cancer** | **Not surprised by anything** | **Form of communication** | **Solutions** |
| --- | --- | --- | --- | --- | --- | --- | --- | --- | --- | --- |
| Female  (16) | 13% (2) | 25% (4) | 6% (1) | 25% (4) | 63% (10) | 19% (3) | 31% (5) | 6%  (1) |  | 6%  (1) |
| Male  (21) | 10% (2) | 25% (5) | 15% (3) |  | 81% (17) | 25% (5) | 5%  (1) | 15% (3) | 15% (3) | 10% (2) |

Table 2E: Results when asked what they learnt, what they took away and what surprised them, by age

| **Age Category** | **Previously unaware of disease** | **Sanitation** | **Symptoms** | **Treatment of disease** | **Transmission /Prevention** | **Cycle of disease** | **Effects of disease e.g. cancer** | **Not surprised by anything** | **Form of communication** | **Solutions** |
| --- | --- | --- | --- | --- | --- | --- | --- | --- | --- | --- |
| <25  (6) |  |  | 33%  (2) |  | 83%  (5) | 50%  (3) |  | 17%  (1) |  |  |
| 25<X<40 (11) |  | 20%  (2) | 10%  (1) |  | 82%  (9) | 20%  (2) | 20%  (2) | 10%  (1) | 10%  (1) | 10%  (1) |
| 40<X<50 (11) | 27%  (3) | 27%  (3) | 9%  (1) | 27%  (3) | 73%  (8) | 18%  (2) | 9%  (1) | 18%  (2) | 9%  (1) |  |
| >50  (9) | 11%  (1) | 44%  (4) |  | 11%  (1) | 56%  (5) | 11%  (1) | 33%  (3) |  | 11%  (1) | 22%  (2) |

### Barriers to elimination – Day 1 and Day 5

| **Key** | | |
| --- | --- | --- |
| Negative % change | 0% change | Positive % change |

Table 3A: Participants were asked to list the perceived barriers to eliminations before (A) and after (B) the intervention (A v B). X% is the percentage change in number of people mentioning an answer per category. This table is divided by occupation.

| **Occupation** | **No barriers** | **Traditional Beliefs** | **Children's activities** | **Lack of Education** | **Lack of Resources** | **People neglecting teaching** | **Travellers urinating in village** |
| --- | --- | --- | --- | --- | --- | --- | --- |
| Farmer  (14) | 0%  (3 v 3) |  |  | 0%  (2 v 2) | -33%  (6 v 4) | 150%  (2 v 5) |  |
| Teacher  (4) |  | -100%  (1 v 0) |  | -50%  (2 v 1) | 100%  (1 v 2) | %  (0 v 1) | %  (0 v 1) |
| CHW (4) | 0%  (1 v 1) |  | -100%  (1 v 0) |  | -100%  (1 v 0) | 100%  (1 v 2) | % (0 v 1) |
| Village Officer (4) |  | -50%  (2 v 1) |  | -100%  (1 v 0) | %  (0 v 1) | 100%  (1 v 2) |  |
| Teenagers  (4) | -50%  (2 v 1) | -100%  (1 v 0) | -100%  (1 v 0) | %  (0 v 1) | -100%  (1 v 0) | %  ( v 2) |  |
| Driver  (2) |  |  |  | 0%  (1 v 1) | 0%  (1 v 1) |  |  |
| Fish Seller /Carpenter (4) | 0%  (1 v 1) |  |  | -100%  (1 v 0) | %  (0 v 1) | 100%  (1 v 2) |  |
| **TOTAL**  **(36)** | **-14% (7 v 6)** | **-75%**  **(4 v 1)** | **-100%**  **(2 v 0)** | **-29%**  **(7 v 5)** | **-10%**  **(10 v 9)** | **180%**  **(5 v 14)** | **%**  **(0 v 2)** |

Table 3B: Change in participants response to perceived barriers to elimination, by village.

| **Village** | **No barriers** | **Traditional Beliefs** | **Children's activities** | **Lack of Education** | **Lack of Resources** | **People neglecting teaching** | **Travellers urinating in village** |
| --- | --- | --- | --- | --- | --- | --- | --- |
| Kigongo  (20) | %  (0 v 1) | -100%  (1 v 0) | -100%  (1 v 0) | -43%  (7 v 4) | -33%  (6 v 4) | 120%  (5 v 11) |  |
| Ng'Wakalima (16) | -29%  (7 v 5) | -67%  (3 v 1) | -100%  (1 v 0) | %  (0 v 1) | 25%  (4 v 5) | %  (0 v 3) | %  (0 v 2) |

Table 3C: Change in participants response to perceived barriers to elimination, by level of education.

| **Level of Education** | **No barriers** | **Traditional Beliefs** | **Children's activities** | **Lack of Education** | **Lack of Resources** | **People neglecting teaching** | **Travellers urinating in village** |
| --- | --- | --- | --- | --- | --- | --- | --- |
| Below primary education (3) |  | -100%  (1 v 0) | -100%  (1 v 0) |  | -50%  (2 v 1) | %  (0 v 2) |  |
| Primary education (23) | 20%  (5 v 6) | -100%  (1 v 0) | -100%  (1 v 0) | -33%  (3 v 2) | -43%  (7 v 4) | 100%  (5 v 10) | %  (0 v 1) |
| Secondary education or above (10) | -100%  (2 v 0) | -50%  (2 v 1) |  | -25%  (4 v 3) | 300%  (1 v 4) | %  (0 v 2) | %  (0 v 1) |

Table 3D: Change in participants response to perceived barriers to elimination, by gender.

| **Gender** | **No barriers** | **Traditional Beliefs** | **Children's activities** | **Lack of Education** | **Lack of Resources** | **People neglecting teaching** | **Travellers urinating in village** |
| --- | --- | --- | --- | --- | --- | --- | --- |
| Female  (16) | -17%  (6 v 5) | -100%  (1 v 0) |  | 0%  (2 v 2) | -60%  (5 v 2) | 250%  (2 v 7) | %  (0 v 1) |
| Male  (20) | 0%  (1 v 1) | -67%  (3 v 1) | -100%  (2 v 0) | -40%  (5 v 3) | 40%  (5 v 7) | 133%  (3 v 7) | %  (0 v 1) |

Table 3E: Change in participants response to perceived barriers to elimination, by age category.

| **Age Category** | **No barriers** | **Traditional Beliefs** | **Children's activities** | **Lack of Education** | **Lack of Resources** | **People neglecting teaching** | **Travellers urinating in village** |
| --- | --- | --- | --- | --- | --- | --- | --- |
| <25  (6) | -50%  (2 v 1) | -100%  (1 v 0) | -100%  (2 v 0) | %  (0 v 1) | -100%  (1 v 0) | 200%  (1 v 3) | %  (0 v 1) |
| 25<X<40  (10) | 0%  (2 v 2) | -50%  (2 v 1) |  | 0%  (2 v 2) | 100%  (2 v 4) | 0%  (1 v 1) | %  (0 v 1) |
| 40<X<50  (11) | 50%  (2 v 3) | -100%  (1 v 0) |  | -50%  (2 v 1) | -50%  (6 v 3) | %  (0 v 4) |  |
| >50  (9) | -100%  (1 v 0) |  |  | -67%  (3 v 1) | 100%  (1 v 2) | 100%   1. v 6) |  |

### Beliefs that intervention could eliminate the disease in the community – Day 5

| **Key** | | |
| --- | --- | --- |
| 0% | 50% | 100% |

Table 4A: Participants beliefs that the intervention could eliminate the disease in the community, by occupation. X% is the percentage of participants in that category giving a response. (#) is the number in that category giving the response.

| **Occupation** | **Method will help** | **Somewhat confident** | **Confident** |
| --- | --- | --- | --- |
| Farmer (14) | 21% (3) | 14% (2) | 64% (9) |
| Teacher (4) | 25% (1) | 50% (2) | 25% (1) |
| Community Health Worker (4) | 50% (2) |  | 50% (2) |
| Village Officer (4) |  | 50% (2) | 50% (2) |
| Teenagers (4) | 25% (1) |  | 75% (3) |
| Driver (3) |  |  | 100% (3) |
| Fish Seller /Carpenter (4) | 50% (2) |  | 50% (2) |
| **TOTAL (37)** | **24% (9)** | **17% (6)** | **59% (22)** |

Table 4B: Participants beliefs that the intervention could eliminate the disease in the community, by village

| **Village** | **Method will help** | **Somewhat confident** | **Confident** |
| --- | --- | --- | --- |
| Kigongo (20) | 45% (9) | 15% (3) | 40% (8) |
| Ng'Wakalima (17) |  | 19% (3) | 82% (14) |

Table 4C: Participants beliefs that the intervention could eliminate the disease in the community, by level of education

| **Level of Education** | **Method will help** | **Somewhat confident** | **Confident** |
| --- | --- | --- | --- |
| Below primary education (3) | 33% (1) | 33% (1) | 33% (1) |
| Primary education (24) | 21% (5) | 13% (3) | 67% (16) |
| Secondary education or above (10) | 30% (3) | 20% (2) | 50% (5) |

Table 4D: Participants beliefs that the intervention could eliminate the disease in the community, by gender

| **Gender** | **Method will help** | **Somewhat confident** | **Confident** |
| --- | --- | --- | --- |
| Female (16) | 25% (4) | 19% (3) | 56% (9) |
| Male (21) | 24% (5) | 15% (3) | 62% (13) |

Table 4E: Participants beliefs that the intervention could eliminate the disease in the community, by age category

| **Age Category** | **Method will help** | **Somewhat confident** | **Confident** |
| --- | --- | --- | --- |
| <25 (6) | 17% (1) |  | 83% (5) |
| 25<X<40 (11) | 27% (3) | 9% (1) | 64% (7) |
| 40<X<50 (11) | 27% (3) | 27% (3) | 45% (5) |
| >50 (9) | 22% (2) | 22% (2) | 56% (5) |

### Knowledge about clean water – Day 1 and 5

Table 5A: Change in clean water knowledge, by occupation

| **Occupation** | **Knowledge stayed the same** | **Said they knew before but now know correctly** | **Did not know before but now knows** | **Knew before and learnt more** |
| --- | --- | --- | --- | --- |
| Farmer (13) | 62% (8) | 8% (1) |  | 31% (4) |
| Teacher (4) | 75% (3) | 25% (1) |  |  |
| Community Health Worker (4) | 50% (2) |  |  | 50% (2) |
| Village Officer (4) | 25% (1) | 25% (1) | 25% (1) | 25% (1) |
| Teenagers (4) | 100% (4) |  |  |  |
| Driver (3) | 33% (1) |  | 33% (1) | 33% (1) |
| Fish Seller /Carpenter (4) | 50% (2) |  |  | 50% (2) |
| **TOTAL (36)** | **58% (21)** | **8% (3)** | **6% (2)** | **28% (10)** |

Table 5B: Change in clean water knowledge, by village

| **Village** | **Knowledge stayed the same** | **Said they knew before but now know correctly** | **Did not know before but now knows** | **Knew before and learnt more** |
| --- | --- | --- | --- | --- |
| Kigongo (20) | 65% (13) |  |  | 35% (7) |
| Ng'Wakalima (16) | 50% (8) | 19% (3) | 13% (2) | 19% (3) |

Table 5C: Change in clean water knowledge, by level of education

| **Level of Education** | **Knowledge stayed the same** | **Said they knew before but now know correctly** | **Did not know before but now knows** | **Knew before and learnt more** |
| --- | --- | --- | --- | --- |
| Below primary education (3) | 67% (2) |  |  | 33% (1) |
| Primary education (23) | 52% (12) | 9% (2) | 4% (1) | 35% (8) |
| Secondary education or above (10) | 70% (7) | 10% (1) | 10% (1) | 10% (1) |

Table 5D: Change in clean water knowledge, by gender

| **Gender** | **Knowledge stayed the same** | **Said they knew before but now know correctly** | **Did not know before but now knows** | **Knew before and learnt more** |
| --- | --- | --- | --- | --- |
| Female (16) | 44% (7) | 13% (2) |  | 44% (7) |
| Male (20) | 70% (14) | 5% (1) | 10% (2) | 15% (3) |

Table 5E: Change in clean water knowledge, by age

| **Age Category** | **Knowledge stayed the same** | **Said they knew before but now know correctly** | **Did not know before but now knows** | **Knew before and learnt more** |
| --- | --- | --- | --- | --- |
| <25 (6) | 100% (6) |  |  |  |
| 25<X<40 (11) | 45% (5) | 18% (2) | 18% (2) | 18% (2) |
| 40<X<50 (11) | 45% (5) | 9% (1) |  | 45% (5) |
| >50 (8) | 63% (5) |  |  | 38% (3) |

### Knowledge about transmission – Day 1 and 5

Table 6A: Change in knowledge about transmission, by occupation.

| **Occupation** | **Knowledge stayed the same** | **Knowledge increased** | **Did not know but now does** |
| --- | --- | --- | --- |
| Farmer (13) |  | 85% (11) | 15% (2) |
| Teacher (4) | 50% (2) | 50% (2) |  |
| Community Health Worker (4) | 25% (1) | 75% (3) |  |
| Village Officer (4) | 50% (2) | 50% (2) |  |
| Teenagers (4) |  | 75% (3) | 25% (1) |
| Driver (3) |  | 67% (2) | 33% (1) |
| Fish Seller /Carpenter (4) |  | 100% (4) |  |
| **TOTAL (36)** | **14% (5)** | **75% (27)** | **11% (4)** |

Table 6B: Change in knowledge about transmission, by village.

| **Village** | **Knowledge stayed the same** | **Knowledge increased** | **Did not know but now does** |
| --- | --- | --- | --- |
| Kigongo (20) | 15% (3) | 80% (16) | 5% (1) |
| Ng'Wakalima (16) | 13% (2) | 69% (11) | 19% (3) |

Table 6C: Change in knowledge about transmission, by level of education

| **Level of Education** | **Knowledge stayed the same** | **Knowledge increased** | **Did not know but now does** |
| --- | --- | --- | --- |
| Below primary education (3) |  | 33% (1) | 67% (2) |
| Primary education (23) | 4% (1) | 87% (20) | 9% (2) |
| Secondary education or above (10) | 40% (4) | 60% (6) |  |

Table 6D: Change in knowledge about transmission, by gender

| **Gender** | **Knowledge stayed the same** | **Knowledge increased** | **Did not know but now does** |
| --- | --- | --- | --- |
| Female (16) | 13% (2) | 81% (13) | 6% (1) |
| Male (20) | 15% (3) | 70% (14) | 15% (3) |

Table 6E: Change in knowledge about transmission, by age

| **Age Category** | **Knowledge stayed the same** | **Knowledge increased** | **Did not know but now does** |
| --- | --- | --- | --- |
| <25 (6) |  | 83% (5) | 17% (1) |
| 25<X<40 (11) | 18% (2) | 73% (8) | 9% (1) |
| 40<X<50 (11) |  | 91% (10) | 9% (1) |
| >50 (8) | 38% (3) | 50% (4) | 13% (1) |

### Knowledge about treatment – Day 1 and 5

Table 7A: Change in knowledge about treatment, by occupation.

| **Occupation** | **Knowledge stayed the same** | **Some knowledge before, now improved** | **Did not know before /knew incorrectly, but now knows correctly** | **Still does not know correctly** | **Used traditional healers before** |
| --- | --- | --- | --- | --- | --- |
| Farmer (13) | 31% (4) | 31% (4) | 23% (3) | 15% (2) | 15% (2) |
| Teacher (4) | 75% (3) |  | 25% (1) |  | 25% (1) |
| Community Health Worker (4) | 50% (2) | 25% (1) | 25% (1) |  | 25% (1) |
| Village Officer (4) | 75% (3) | 25% (1) |  |  | 25% (1) |
| Teenagers (4) |  | 25% (1) | 50% (2) | 25% (1) |  |
| Driver (3) |  |  | 100% (3) |  |  |
| Fish Seller /Carpenter (4) | 25% (1) | 25% (1) | 50% (2) |  | 25% (1) |
| **TOTAL (36)** | **36% (13)** | **22% (8)** | **33% (12)** | **8% (3)** | **17% (6)** |

Table 7B: Change in knowledge about treatment, by village.

| **Village** | **Knowledge stayed the same** | **Some knowledge before, now improved** | **Did not know before /knew incorrectly, but now knows correctly** | **Still does not know correctly** | **Used traditional healers before** |
| --- | --- | --- | --- | --- | --- |
| Kigongo (20) | 30% (6) | 30% (6) | 40% (8) |  | 10% (2) |
| Ng'Wakalima (16) | 44% (7) | 13% (2) | 25% (4) | 19% (3) | 25% (4) |

Table 7C: Change in knowledge about treatment, by level of education

| **Level of Education** | **Knowledge stayed the same** | **Some knowledge before, now improved** | **Did not know before /knew incorrectly, but now knows correctly** | **Still does not know correctly** | **Used traditional healers before** |
| --- | --- | --- | --- | --- | --- |
| Below primary education (3) |  |  | 67% (2) | 33% (1) | 33% (1) |
| Primary education (23) | 35% (8) | 26% (6) | 35% (8) | 4% (1) | 17% (4) |
| Secondary education or above (10) | 50% (5) | 20% (2) | 20% (2) | 10% (1) | 10% (1) |

Table 7D: Change in knowledge about treatment, by gender.

| **Gender** | **Knowledge stayed the same** | **Some knowledge before, now improved** | **Did not know before /knew incorrectly, but now knows correctly** | **Still does not know correctly** | **Used traditional healers before** |
| --- | --- | --- | --- | --- | --- |
| Female (16) | 38% (6) | 25% (4) | 31% (5) | 6% (1) | 19% (3) |
| Male (20) | 35% (7) | 20% (4) | 35% (7) | 10% (2) | 15% (3) |

Table 7E: Change in knowledge about treatment, by age.

| **Age Category** | **Knowledge stayed the same** | **Some knowledge before, now improved** | **Did not know before /knew incorrectly, but now knows correctly** | **Still does not know correctly** | **Used traditional healers before** |
| --- | --- | --- | --- | --- | --- |
| <25 (6) | 17% (1) | 33% (2) | 33% (2) | 17% (1) |  |
| 25<X<40 (11) | 36% (4) | 18% (2) | 45% (5) |  | 9% (1) |
| 40<X<50 (11) | 27% (3) | 18% (2) | 45% (5) | 9% (1) | 36% (4) |
| >50 (8) | 63% (5) | 25% (2) |  | 13% (1) | 13% (1) |

# Perception to involvement in schistosomiasis elimination

Table 8A: Changes in perception to involvement in schistosomiasis elimination, by occupation.

| **Occupation** | **Same involvement** | **Not involved before but will now be involved** | **Said they will educate others** |
| --- | --- | --- | --- |
| Farmer (14) | 21% (3) | 79% (11) | 29% (4) |
| Teacher (4) | 75% (3) | 25% (1) | 25% (1) |
| Community Health Worker (4) | 50% (2) | 50% (2) | 75% (3) |
| Village Officer (4) | 50% (2) | 50% (2) | 25% (1) |
| Teenagers (4) | 25% (1) | 75% (3) | 25% (1) |
| Driver (3) |  | 100% (3) |  |
| Fish Seller /Carpenter (4) |  | 100% (4) | 25% (1) |
| **TOTAL (37)** | **30% (11)** | **70% (26)** | **30% (11)** |

Table 8B: Changes in perception to involvement in schistosomiasis elimination, by village.

| **Village** | **Same involvement** | **Not involved before but will now be involved** | **Said they will educate others** |
| --- | --- | --- | --- |
| Kigongo (20) | 25% (5) | 75% (15) | 20% (4) |
| Ng'Wakalima (17) | 35% (6) | 65% (11) | 41% (7) |

Table 8C: Changes in perception to involvement in schistosomiasis elimination, by level of education.

| **Level of Education** | **Same involvement** | **Not involved before but will now be involved** | **Said they will educate others** |
| --- | --- | --- | --- |
| Below primary education (3) | 33% (1) | 67% (2) |  |
| Primary education (24) | 21% (5) | 79% (19) | 38% (9) |
| Secondary education or above (10) | 50% (5) | 50% (5) | 20% (2) |

Table 8D: Changes in perception to involvement in schistosomiasis elimination, by gender.

| **Gender** | **Same involvement** | **Not involved before but will now be involved** | **Said they will educate others** |
| --- | --- | --- | --- |
| Female (16) | 31% (5) | 69% (11) | 38% (6) |
| Male (21) | 29% (6) | 71% (15) | 24% (5) |

Table 8E: Changes in perception to involvement in schistosomiasis elimination, by age.

| **Age Category** | **Same involvement** | **Not involved before but will now be involved** | **Said they will educate others** |
| --- | --- | --- | --- |
| <25 (6) | 17% (1) | 83% (5) | 50% (3) |
| 25<X<40 (11) | 27% (3) | 73% (8) | 27% (3) |
| 40<X<50 (11) | 27% (3) | 73% (8) | 27% (3) |
| >50 (9) | 44% (4) | 56% (5) | 22% (2) |

#

# B. PLAY/FILM AUDIENCE INTERVIEW RESPONSES

Table 9A: Interview answers averaged according to watching the play or film, % is percentage of people within a category who mentioned a topic and (#) is the number of people who mentioned it

| **Play or film?** | **Urination location** | **Government bring clinic /water** | **Go to hospital** | **Water contact** | **Effects of schistosomiasis** | **Sanitation/clean environment** | **Use clean water** | **Symptoms** | **Educate others** | **Transmission** | **Learnt something incorrect** |
| --- | --- | --- | --- | --- | --- | --- | --- | --- | --- | --- | --- |
| Play (39) | 54% (21) | 21% (8) | 21% (8) | 31% (12) | 8%  (3) | 36% (12) | 38% (15) | 21% (8) | 21% (8) | 18% (7) | 5% (2) |
| Film (20) | 45% (9) | 10% (2) | 30% (6) | 80% (16) | 10% (2) | 30% | 35% (7) | 20% (4) | 10% (2) | 20% (4) | 10% (2) |

Table 9B: Play/film interview answers on learnings, by gender.

| **Gender** | **Urination location** | **Government bring clinic /water** | **Go to hospital** | **Water contact** | **Sanitation/clean environment** | **Use clean water** | **Symptoms** | **Educate others** | **Transmission** | **Construct toilets** | **Collect water in early morning/evening** | **Average number of learning topics** |
| --- | --- | --- | --- | --- | --- | --- | --- | --- | --- | --- | --- | --- |
| Female (37) | 43% (16) | 19% (7) | 19% (7) | 41% (15) | 30% (11) | 41% (15) | 19% (7) | 14% (5) | 19% (7) | 11% (4) | 22% (8) | 2.95 |
| Male (22) | 64% (14) | 14% (3) | 32% (7) | 59% (13) | 41% (9) | 32% (7) | 23% (5) | 23% (5) | 18% (4) |  | 5% (1) | 3.23 |
| **TOTAL (59)** | **51% (30)** | **17% (10)** | **24% (14)** | **47% (28)** | **34% (20)** | **37% (22)** | **20% (12)** | **17% (10)** | **19% (11)** | **7%**  **(4)** | **15% (9)** | **3.05** |

Table 9C: Play/film interview answers by village (all topics).

| **Village** | **Urination location** | **Government bring clinic /water** | **Go to hospital** | **Water contact** | **Effects of schistosomiasis** | **Sanitation /clean environment** | **Use clean water** |
| --- | --- | --- | --- | --- | --- | --- | --- |
| Chole  (10) | 50% (5) |  | 40% (4) | 70% (7) | 10% (1) | 50% (5) | 50% (5) |
| Nyang Holongo (10) | 40% (4) | 20% (2) | 20% (2) | 90% (9) | 10% (1) | 10% (1) | 20% (2) |
| Ng'Wakalima (17) | 76% (13) | 41% (7) | 29% (5) | 18% (3) |  | 12% (2) | 35% (6) |
| Kigongo  (22) | 36% (8) | 5% (1) | 14% (3) | 41% (9) | 14% (3) | 55% (12) | 41% (9) |

| **Village** | **Symptoms** | **Educate others** | **Did not know disease before** | **Transm-ission** | **Learnt something incorrect** | **Construct toilets** | **Collect water at good time** | **Wear shoes to the toilet** |
| --- | --- | --- | --- | --- | --- | --- | --- | --- |
| Chole  (10) | 30% (3) | 10% (1) |  |  | 10% (1) |  |  |  |
| Nyang Holongo (10) | 10% (1) | 10% (1) |  | 40% (4) | 10% (1) |  |  |  |
| Ng'Wakalima (17) | 29% (5) | 24% (4) | 6% (1) | 24% (4) | 12% (2) |  |  |  |
| Kigongo  (22) | 14% (3) | 18% (4) |  | 14% (3) |  | 18% (4) | 41% (9) | 9% (2) |
