# Supplementary material for: A novel theatre-based behaviour change approach for influencing community uptake of schistosomiasis control measures
Source: Parasit Vectors. 2022 Aug 25;15:301. doi: 10.1186/s13071-022-05421-5 (PMC9406251; doi:10.1186/s13071-022-05421-5)
Supplement: Supplementary file 1 — Additional file 1: Text S1. Qualitative interviews and focus group discussions topic guide and questions. Text S2. Acting for Health methodology. Table S1. Intervention workshop cohort and drama and film audience survey responses. Table S2. Emergent themes and narrative quotations from formative qualitative findings. Table S3. Quantitative questionnaire survey results for baseline and post intervention for Tanzania. Table S4. Quantitative questionnaire survey results for baseline and post intervention for Ethiopia. [file 13071_2022_5421_MOESM1_ESM.zip › Table S2.docx]

**Table S2**: Emergent themes and narrative quotations from qualitative findings to explore schistosomiasis risk awareness, attitude, perception, practices, and knowledge on transmission and control measures

|  | **Narrative quotations** |
| --- | --- |
| **Risk awareness** | *Male FGD*: “Bilharzia is a big problem because some people boil water for drinking, others drink water without boiling, wherever they find water even if it is in the bush when they go to collect firewood, they just drink in order to quench their thirst. He/she acquires bilharzias directly and this problem shall never end because people use water rampantly.”; “Personally I think Schistosomiasis is due to not being careful with hygiene at our home, for example us around here who are living close to the Lake, you would find I have gone to take bath at the Lake, while bathing I will just urinate in the same water and planting the parasites. If you go to our homes the hygiene isn't there at all, they aren't being cleaned, when the woman will come to fetch water, when she gets home she would just pour it in the storing dish without considering the effects of boiling it, she is just keeping it as she brought it.” |
| **Attitude and perceptions on severity** | *Female FGD*: “And for the side of women, we are suffering from lower stomach pain, we think that it is U.T.I. Another thing which disturbs women is exceeding days of bleeding during menstruation period.”; “Health problems which disturb us frequently in the community are typhoid, U.T.I diarrhoea, vomiting and Malaria.”; “The most common health problem which disturbs our community is bilharzia, children several times pass blood during urination.”; “We can rank malaria, there is typhoid and UTI and bilharzia disease followed”  *Male FGD*: “When we talk about common diseases in our community, Typhoid and amoebic dysentery, they are most leading than Bilharzia, the most problem is typhoid, amoeba dysentery and sexually transmitted diseases.”; “You know that Bilharzia is affecting us slowly, if you mobilize people for testing, I am sure everyone will have Bilharzia, it is just that it affects slowly unlike other diseases like malaria.”; “I suffered from this disease when I was in standard one until I reached standard five. I was suffering from stomach pain and discharging blood in the urine, also pain during urination.”; “I was very sick but by the time when I was fifteen years the disease stopped, and I was not urinating blood anymore.”; “I know at the time I was at school we were taught that water from the rivers are safe because they are flowing water because they flow with the bacteria in it. We were taught that any flowing water is safe.” |
| **Knowledge on transmission** | *Female FGD*: “For us women, when we urinate, we use to squat so when an infected person has urinated and another goes to squat and urinate there, she may acquire bilharzia because the bacteria have steam which enables them to come up to you and can be transmitted that way.”; “Bilharzias can be caused by sexual intercourse. If one partner has it, he/she can transmit to the other.”  *Male FGD*: “In latrines, someone may defecate, another person will come and defecate, therefore the germs will enter my body because the one that has defecated there has the disease, even in a bathroom if I bath and urinate there someone else must get the disease.”; “In the past schistosomiasis was spreading through clans.”; “If I have schistosomiasis and I have a wife, when I give birth to children they will surely have it, that's how it was. When you give birth to the children, after they have grown up they will start urinating blood because I had it. If the father has it then even the children must have.”; “You would find someone is eating food with too much salt then you would find he is acquiring that disease.”; “Yes, I have seen the media about seminars on agricultural groups, the vegetable growers, how they are affected by bilharzia, because bilharzia are worms found through snails…After watching that information (news) on the TV, they were showing that, when you have contact the water for a long time, your skin becomes wet and expand and allow germs which cause bilharzia to penetrate, so if you are going to fetch water, fetch water from 6 pm to 7pm when those worms will be absent around the Lake shore. The worms will for those hours be far away because the water is cool. The worms come at the Lake shore by heat to get food.”; “Our bathroom has no cement floor, if I as the first person going to bath is already a victim of bilharzia, I will urinate at the bath, the baby will go bathing as a second person, he will contact with the urine.”; “If you observe it is mostly caused by poor water infrastructures because the water that we are drinking is from the wells and not from the tap. Most of us drink water from the wells and it is dirty.” |
| **Treatment seeking behaviour** | *Female FGD*: “Many people don’t know that bilharzia can be treated in the hospital so they use traditional medicines, they just run and use traditional medicine, and they get a lot of effects and people can lose their lives.”; “In the program of distributing medicines at schools, it is until the parents are educated about these medicines, because some of the parents refuse that medicines and complain that the children will die, so education is not there. If they will be educated, the system will be continuous.”; “The drug services are provided to the kids only, those medications are not reaching us and most of us are far from hospitals.”; “I know someone who was sick and I haven’t seen him using the medicine from the doctors, he only used the local medicine and he recovered.”  *Male FGD*: “Bilharzia is transmitted here in our village because of environment, and we don’t have education. If we could be educated, we could have awareness on how Bilharzia is transmitted. Here we are silent, it is just entering us silently, when you go to Bukumbi hospital you are told you have Bilharzia, but here in the village you will be told it is witchcraft.”; “Some children who have been given tablets without being tested and maybe had allergy and the drug has severely affected them, while others are losing consciousness, others get this rashes and this causes parents to prevent their children to go to school when they hear that pupils will be given tablets.”; “I have my brother who was treated traditionally and you may not believe it. He was suffering from bilharzia for twenty-five years. We found a traditional doctor at Kasamwa Geita region. if you know that place. When we arrived there, he told us to find two kilograms of sugar, five litres of water, in fact the sugar for that period was seven hundred shillings per kilogram, we bought two kilograms for one thousand four hundred shillings, water was free, we sent to the traditional doctor. He told us to pay five thousand and it was in nineteen eighty six year, and we gave him five thousand shillings. He continued to urinate with full blood only and the traditional doctor said because he had many years of the disease. If you finish the five litres of medicine, you'll be completely safe, and the truth is; until today my brother has survived, now that's why I was saying that in previous years elders knew the medicine.” |
| **Control** | *Female FGD*: “There isn’t any way for preventing bilharzia so that you won't acquire it again, only the medicine will help prevent that disease. I don't see any other way simply because if we could have done something before then we wouldn't be suffering from bilharzia.”; “They usually bring the medicine at school and we prepare the kids to take the medicine for bilharzia, but they will get better for a short period of time like three months only. If they go for testing again, they are found with it, so it has become a normal thing and the water is not clean.”  *Male KII:* “I think that the best way is toilets. This way will also protect us from so many things, and it will be treatment for curing many diseases affecting the stomach. For that reason, I support this method, in fact it would be the first of all from other methods.”  *Male FGD:* “I think in order to prevent this bilharzia from spreading, we should make our environment clean, ensure hygiene in our communities.”; “The provision of clean and safe water is the prevention.”. |
| **Self-regulation and Responsibility** | *Male KII*: “In order to eliminate schistosomiasis, government must construct water infrastructure to reduce the number of people who are going to the lake.”  *Male FGD*: “For the side of farmers, I am asking government to think and look to minimize the price of those equipment which protect people from acquiring bilharzia. For example, as I am speaking if you come to Moshi and enquire. I should wear long gloves and other things. I went to the agriculture inputs shop and found those waterproof gumboots which their size reach here and are tight, that raincoat which is made of plastic to prevent water penetration, and they told me its price is three hundred sixty thousand shillings. I have five youths, including me we are six, how can I afford that cost to protect ourselves from bilharzia.”; “I think a big challenge there is money, that will be the first challenge because that water project cannot be there without money so the first thing is money. The second thing is us the community, if we stay together and find someone in government who will come to help on the issue of clean water, as the community we have to participate.”  *Female FGD*: “I can contribute five thousand shillings per month for water service.”; “According to my financial capacity I can contribute three thousand shillings per month.” |
