# Supplementary material for: A novel theatre-based behaviour change approach for influencing community uptake of schistosomiasis control measures
Source: Parasit Vectors. 2022 Aug 25;15:301. doi: 10.1186/s13071-022-05421-5 (PMC9406251; doi:10.1186/s13071-022-05421-5)
Supplement: Supplementary file 1 — Additional file 1: Text S1. Qualitative interviews and focus group discussions topic guide and questions. Text S2. Acting for Health methodology. Table S1. Intervention workshop cohort and drama and film audience survey responses. Table S2. Emergent themes and narrative quotations from formative qualitative findings. Table S3. Quantitative questionnaire survey results for baseline and post intervention for Tanzania. Table S4. Quantitative questionnaire survey results for baseline and post intervention for Ethiopia. [file 13071_2022_5421_MOESM1_ESM.zip › Table S3.docx]

**Tanzania**

**Table S3**: Summary of Tanzania survey results for Baseline (BL) and Post-intervention (PI) with the effects of gender, age, educational level and the different intervention groups. Neutral+ change means there was no difference post intervention but still a desired outcome; Negative* means a significant increase or decrease post intervention but not a desired outcome; Positive means there was a significant increase or decrease post intervention, and it was the desired outcome

|  | **Yes /selected option**  **BL %** | **Yes/ selected option**  **PI %** | **Paired t-test**  **N = 522**  ***p*-Value** | **Positive, negative* or neutral+ change** | **Gender effect, chi-square *p*-Value** | | **Age effect, chi-square *p*-Value** | | **Education effect, chi-square**  ***p*-Value** | | **Effect of intervention groups** | | | | |
| --- | --- | --- | --- | --- | --- | --- | --- | --- | --- | --- | --- | --- | --- | --- | --- |
|  |  |  |  |  | **BL** | **PI** | **BL** | **PI** | **BL** | **PI** | **Grp 1 N=104**  **%(n)** | **Grp 2**  **N=13**  **% (n)** | **Grp 3 N=170**  **% (n)** | **Grp 4 N=382**  **% (n)** | ***p-*Value** |
| **I. Risk factors: Knowledge and awareness of schistosomiasis danger** | | | | | | | | | | | | | | | |
| a. Have you ever heard of schistosomiasis or bilharzia? | 98% | 99% | 0.1318 | Neutral+ | 0.746 | 0.031 | - | - | 0.476 | 0.741 | - | - | - | - | - |
| b. Do diseases affect the children or the adults?   1. Children 2. Adults 3. Both | 3%  9%  88% | 2%  3%  95% | 0.0003 | Positive | 0.806 | 0.840 | 0.484 | 0.879 | 0.516 | 0.930 | 1% (1)  9% (9)  90% (94) | 8% (1)  0 (0)  92% (12) | 1% (2)  2% (4)  97% (164) | 3% (11)  2% (8)  95% (363) | 0.31 |
| c. Do you think schistosomiasis is dangerous?   1. Very dangerous 2. Dangerous 3. Slightly dangerous 4. Not dangerous | 46%  43%  9%  2% | 51%  45%  4%  0 | 0.0002 | Positive | 0.007 | 0.160 | 0.269 | 0.197 | 0.084 | 0.334 | 51% (53)  46% (48)  3% (3)  0 (0) | 23% (3)  77% (10)  0 (0)  0 (0) | 56% (95)  40% (68)  4% (6)  0 (1) | 49% (186)  47% (178)  4% (17)  0 (1) | 0.417 |
| d. If your child ever had blood in his/her urine would this be worrying to you | 98% | 99% | 0.3662 | Neutral+ | 0.214 | 0.788 | - | - | 0.994 | 0.738 | - | - | - | - | - |
| **II. Risk factors: Awareness of schistosomiasis transmission** | | | | | | | | | | | | | | | |
| a. Can you get schistosomiasis by drinking dirty water? | 87% | 92% | 0.0058 | Negative* | 0.181 | 0.036 | 0.044 | 0.253 | 0.024 | 0.024 | 91% (95) | 85% (11) | 94% (160) | 92% (352) | 0.036 |
| b. Can you get schistosomiasis by contact with infested water in river, lakes and ponds? | 84% | 94% | 0.0000 | Positive | 0.083 | 0.267 | 0.075 | 0.235 | 0.001 | 0.015 | 99% (103) | 100% (13) | 96% (164) | 91% (349) | 0.023 |
| c. How are people spreading the disease?   1. By urinating in the water body 2. By human faeces in the water body 3. Humans do not spread the disease 4. Don’t know 5. Other | 38%  4%  2%  19%  37% | 18%  47%  1%  12%  22% | 0.000 | Positive | 0.877 | 0.284 | 0.356 | 0.033 | 0.217 | 0.341 | 13% (14)  41% (43)  2% (2)  13% (13)  31% (32) | 31% (4)  46% (6)  0 (0)  15% (2)  8% (1) | 21% (35)  51% (86)  0 (0)  10% (18)  18% (31) | 17% (66)  47% (176)  1% (5)  12% (47)  23% (86) | 0.387 |
| **III. Attitude factors: Treatment seeking behaviour** |  |  |  |  |  |  |  |  |  |  |  |  |  |  |  |
| a. Does an infected person need treatment? | 100% | 100% | - | Neutral+ | - | - | - | - | - | - | - | - | - | - | - |
| b. Where is the right place to seek treatment?   1. Health facility/centre/hospital 2. Drug shop 3. Traditional healer 4. Other | 95%  3%  1%  1% | 99%  1%  0%  0% | 0.0006 | Positive | 0.233 | 0.505 | 0.751 | 0.784 | 0.303 | 0.695 | 99% (103)  1% (1)  0 (0)  0 (0) | 92% (12)  8% (1)  0 (0)  0 (0) | 100% (170)  0 (0)  0 (0)  0 (0) | 99% (378)  1% (3)  0 (1)  0 (0) | 0.103 |
| c. Have you or your child ever been treated for schistosomiasis before? | 49% | 61% | 0.0000 | Positive | 0.000 | 0.000 | 0.000 | 0.056 | 0.746 | 0.003 | 68% (71) | 77% (10) | 62% (105) | 58% (222) | 0.167 |
| **IV. Norm and contextual factors: Community control and prevention of schistosomiasis** | | | | | | | | | | | | | | | |
| a. How can schistosomiasis be controlled or prevented?   1. Mass administration of drugs (praziquantel) 2. Disposal of faeces in toilets/latrines 3. Provision and use of safe water sources 4. Minimising or avoiding contact with infested waters 5. Snail control (molluscicides) 6. All of the above | 48%  13%  59%  27%  1%  0% | 40%  21%  72%  39%  2%  0% | 0.0028  0.0000  0.000  0.0000  0.1657  1.000 | Negative*  Positive  Positive  Positive  Neutral+  Neutral+ | 0.989  0.547  0.035  0.984  -  - | 0.210  0.870  0.223  0.491  -  - | 0.081  0.098  0.098  0.489  -  - | 0.683  0.648  0.322  0.069  -  - | 0.000  0.01  0.011  0.000  -  - | 0.000  0.328  0.000  0.000  -  - | 45% (47)  30% (31)  78% (81)  29% (30)  2% (2)  2% (2) | 23% (3)  15% (2)  85% (11)  62% (8)  8% (1)  0 (0) | 42% (71)  24% (40)  78% (132)  40% (67)  4% (6)  0 (0) | 39% (149)  18% (68)  68% (260)  40% (154)  1% (5)  0 (0) | 0.384  0.044  0.037  0.056  0.182  0.012 |
| b. What obstacles do you think will be faced when providing these interventions?   1. Lack of money 2. Lack of health facilities/hospital 3. Lack of awareness/education 4. Lack of sufficient medicine 5. Lack of cooperation/cultural limitations 6. Lack of water infrastructure & government support 7. Other 8. No obstacles 9. I don’t know | 5%  13%  11%  1%  15%  2%  3%  40%  10% | 11%  4%  12%  4%  12%  38%  4%  6%  9% | 0.0000 | Positive | 0.003 | 0.108 | 0.804 | 0.553 | 0.000 | 0.507 | 9% (9)  6% (6)  13% (13)  4% (4)  14% (14)  42% (42)  3% (3)  4% (4)  5% (5) | 42% (5)  0 (0)  0 (0)  8% (1)  17% (2)  25% (3)  0 (0)  8% (1)  0 (0) | 9% (15)  3% (6)  12% (20)  4% (7)  15% (25)  40% (67)  5% (8)  8% (13)  4% (7) | 12% (47)  3% (13)  12% (45)  5% (18)  10% (37)  36% (140)  5% (19)  5% (19)  12% (45) | 0.078 |
| c. Which interventions are you willing to pay for, if need be?   1. Use of clean water 2. Built toilets/ infrastructure 3. Not ready to contribute 4. Any future plan 5. Medicine 6. Build health facility/hospital 7. Other 8. Education/ Awareness | 41%  10%  3%  2%  3%  36%  3%  2% | 22%  41%  2%  4%  12%  11%  7%  1% | 0.0033 | Positive | 0.184 | 0.117 | 0.781 | 0.097 | 0.091 | 0.000 | 25% (26)  41% (42)  1% (1)  3% (3)  13% (14)  13% (14)  4% (4)  0 (0) | 34% (4)  42% (5)  8% (1)  0 (0)  8% (1)  0 (0)  8% (1)  0 (0) | 23% (39)  41% (69)  1% (1)  2% (3)  15% (26)  11% (19)  6% (11)  1% (2) | 21% (80)  41% (158)  2% (7)  5% (19)  11% (42)  10% (38)  8% (32)  2% (7) | 0.547 |
| **V. Ability and contextual factors: Water contact behaviours** | |  |  |  |  |  |  |  |  |  |  |  |  |  |  |
| a. What disease (if any) can you get from your water source for domestic, recreational and occupational use?   1. Schistosomiasis 2. Others 3. I don’t know 4. No disease | 57%  25%  12%  6% | 85%  14%  1%  0 | 0.000 | Positive | 0.037 | 0.097 | 0.285 | 0.079 | 0.000 | 0.032 | 89% (89)  11% (11)  0 (0)  0 (0) | 92% (11)  8% (1)  0 (0)  0 (0) | 86% (145)  14% (23)  0 (0)  0 (0) | 84% (312)  14% (54)  1% (4)  1% (3) | 0.682 |
| b. Why do you use this water source for domestic, recreational and/or occupational needs?   1. Convenience 2. Only available source 3. Affordable 4. Privacy 5. Social activity | 44%  35%  10%  0%  11% | 57%  67%  10%  1%  11% | 0.0000  0.0000  0.8233  0.1800  0.8447 | Negative*  Negative*  Neutral  Neutral  Neutral | 0.239  0.206  -  -  - | 0.699  0.126  -  -  - | 0.098  0.816  -  -  - | 0.479  0.150  -  -  - | 0.879  0.867  -  -  - | 0.174  0.150  -  -  - | 57% (59)  62% (64)  -  -  - | 77% (10)  54% (7)  -  -  - | 58% (99)  76% (130)  -  -  - | 56% (217)  64% (247)  -  -  - | 0.521  0.014  -  -  - |
| c. Will you avoid/minimise all existing contact with contaminated water if you have an alternative clean water source? | 94% | 94% | 0.3178 | Neutral | 0.589 | 0.660 | 0.029 | 0.970 | 0.480 | 0.018 | - | - | - | - | - |
| d. Give reasons to why you will avoid/minimise all existing contact with contaminated water   1. Since I will have clean and safe water 2. We will be disease free 3. Convenience and proximity 4. I can’t avoid water contact | 77%  17%  2%  4% | 50%  38%  9%  3% | 0.0000 | Positive | 0.273 | 0.494 | 0.150 | 0.118 | 0.608 | 0.241 | 43% (45)  42% (44)  11% (11)  4% (4) | 73% (8)  18% (2)  9% (1)  0 (0) | 54% (86)  37% (59)  6% (10)  3% (4) | 50% (178)  37% (133)  9% (34)  4% (13) | 0.647 |
| **VI. Self-regulation and contextual factors: Responsibility** | |  |  |  |  |  |  |  |  |  |  |  |  |  |  |
| a. Who should buy/install water infrastructure?   1. Government 2. Community fund 3. Individuals 4. Other (NGOs, community leader) | 71%  7%  7%  15% | 72%  18%  5%  5% | 0.0001 | Positive | 0.007 | 0.968 | 0.565 | 0.389 | 0.296 | 0.395 | 68% (71)  16% (17)  7% (7)  9% (9) | 100% (13)  0% (0)  0% (0)  0% (0) | 70% (119)  20% (34)  7% (12)  3% (5) | 72% (277)  18% (67)  4% (16)  6% (22) | 0.088 |
| b. Who should maintain the installed water system?   1. Government 2. Community 3. Individuals/users 4. Other (NGOs, community leader) | 13%  73%  4%  10% | 57%  9%  19%  15% | 0.0005 | Positive | 0.001 | 0.966 | 0.066 | 0.026 | 0.145 | 0.447 | 63% (65)  3% (3)  15% (16)  19% (20) | 77% (10)  0 (0)  23% (3) | 59% (100)  12% (21)  18% (30)  11% (19) | 54% (205)  10% (38)  20% (76)  16% (63) | 0.059 |
| c. Let’s imagine that in 5 years there is no more schistosomiasis (bilharzia), How could you achieve this?   1. Expansion of health services/centres 2. Enhancing community 3. Improve sanitation and water services 4. Avoid water contact 5. Mass drug administration 6. Increase awareness/education 7. Other | 6%  3%  62%  1%  18%  8%  2% | 4%  1%  64%  4%  12%  12%  3% | 0.1981 | Neutral | 0.195 | 0.013 | - | - | 0.022 | 0.001 | - | - | - | - | - |
| d. Has your behaviour and attitude changed as a result of this interventions? | - | 80% (249) | - | - | - | 0.512 | - | 0.149 | - | 0.368 | 84% (71) | 92% (12) | 83% (136) | 61% (30) | 0.0004 |
| e. How has your behaviour changed?  i. I have built a latrine and use it and I no longer openly defecate or urinate  ii. I avoid lake/pond water and now use clean water from closed well, tube well, rainwater harvesting  iii. I am no longer bathing or swimming in lake or pond  iv. I have stopped my children from playing or swimming in lake or pond  v. I now take medication  vi. I have learnt much (*nothing specific mentioned and so we could assume no change here)  vii. I use gumboots  viii. I have not changed because of my work  ix. I have not changed because we still have the same contaminated water source | -  -  -  -  -  -  -  -  - | 33% (83)  52% (130)  17% (42)  10% (26)  4% (9)  20% (51)  11% (7)  5% (3)  68% (42) |  |  |  |  |  |  |  |  | 38% (27)  56% (40)  14% (10)  11% (8)  6% (4)  17% (12)  1% (1)  0 (0)  79% (11) | 58% (7)  42% (5)  25% (3)  8% (1)  0 (0)  8% (1)  8% (1)  0 (0)  100% (1) | 30% (41)  49% (67)  15% (20)  9% (12)  4% (5)  24% (32)  3% (4)  11% (3)  89% (25) | 27% (8)  60% (18)  30% (9)  17% (5)  0 (0)  20% (6)  3% (1)  0 (0)  26% (5) |  |
