# Supplementary material for: A novel theatre-based behaviour change approach for influencing community uptake of schistosomiasis control measures
Source: Parasit Vectors. 2022 Aug 25;15:301. doi: 10.1186/s13071-022-05421-5 (PMC9406251; doi:10.1186/s13071-022-05421-5)
Supplement: Supplementary file 1 — Additional file 1: Text S1. Qualitative interviews and focus group discussions topic guide and questions. Text S2. Acting for Health methodology. Table S1. Intervention workshop cohort and drama and film audience survey responses. Table S2. Emergent themes and narrative quotations from formative qualitative findings. Table S3. Quantitative questionnaire survey results for baseline and post intervention for Tanzania. Table S4. Quantitative questionnaire survey results for baseline and post intervention for Ethiopia. [file 13071_2022_5421_MOESM1_ESM.zip › Table S4.docx]

**Ethiopia**

**Table S4**: Summary of Ethiopia survey results for Baseline (BL) and Post-intervention (PI) with the effects of gender, age and educational level. Neutral+ change means there was no difference post intervention but still a desired outcome; Negative* means a significant increase or decrease post intervention but not a desired outcome; Positive means there was a significant increase or decrease post intervention, and it was the desired outcome

|  | **Yes /selected option**  **BL %** | **Yes/ selected option**  **PI %** | **Paired t-test**  **N = 369**  ***p*-Value** | **Positive, negative* or neutral+ change** | **Gender effect, chi-square *p*-Value** | | **Age effect, chi-square *p*-Value** | | **Education effect, chi-square, *p*-Value** | |
| --- | --- | --- | --- | --- | --- | --- | --- | --- | --- | --- |
|  |  |  |  |  | **BL** | **PI** | **BL** | **PI** | **BL** | **PI** |
| **I. Risk factors: Knowledge and awareness of schistosomiasis danger** | | | | | | | | | | |
| a. Have you ever heard of schistosomiasis or bilharzia? | 94% | 81% | 0.0000 | Negative* | 0.533 | 0.539 | 0.851 | 0.919 | 0.174 | 0.069 |
| b. Do diseases affect the children or the adults?   1. Children 2. Adults 3. Both | 14%  11%  75% | 10%  7%  83% | 0.0048 | Positive | 0.082 | 0.545 | 0.002 | 0.495 | 0.342 | 0.912 |
| c. Do you think schistosomiasis is dangerous?   1. Very dangerous 2. Dangerous 3. Slightly dangerous 4. Not dangerous | 50%  44%  4%  2% | 66%  27%  3%  4% | 0.0219 | Positive | 0.000 | 0.251 | 0.000 | 0.373 | 0.00 | 0.229 |
| d. If your child ever had blood in his/her urine would this be worrying to you? | 99% | 99% | 0.771 | Neutral+ | 0.367 | 0.410 | - | - | 0.687 | 0.688 |
| **II. Risk factors: Awareness of schistosomiasis transmission** | | | | | | | | | | |
| 1. Can you get schistosomiasis by drinking dirty water? | 75% | 85% | 0.000 | Negative* | 0.418 | 0.844 | 0.095 | 0.469 | 0.014 | 0.184 |
| 1. Can you get schistosomiasis by contact with infested water in river, lakes and ponds? | 94% | 97% | 0.0222 | Positive | 0.792 | 0.300 | 0.122 | 0.710 | 0.040 | 0.324 |
| c. How are people spreading the disease?  i. By urinating in the water body  ii. By human faeces in the water body  iii. Humans do not spread the disease  iv. Don’t know | 27%  10%  36%  27% | 68%  14.5%  14%  3.5% | 0.0000 | Positive | 0.032 | 0.717 | 0.000 | 0.398 | 0.000 | 0.132 |
| **III. Attitude factors: Treatment seeking behaviour** | | | | | | | | | | |
| a. Does an infected person need treatment? | 98% | 97% | 0.17 | Neutral+ | 0.255 | 0.664 | 0.161 | 0.588 | 0.703 | 0.313 |
| b. Where is the right place to seek treatment?   1. Health facility/centre/hospital 2. Drug shop 3. Traditional healer 4. Religious centre 5. Other | 95%  4%  0.5%  -  0.5% | 98%  2%  0%  -  - | 0.0195 | Positive | 0.581 | 0.541 | 0.000 | 0.645 | 0.160 | 0.217 |
| c. Have you or your child ever been treated for schistosomiasis before? | 27% | - | - | - | - | - | - | - | - | - |
| **IV. Norm and contextual factors: Community control and prevention of schistosomiasis** | | | | | | | | | | |
| a. How can schistosomiasis be controlled or prevented?  i. Mass administration of drugs (praziquantel)  ii. Disposal of faeces in toilets/latrines  iii. Provision and use of safe water sources  iv. Minimising or avoiding contact with infested waters  v. Snail control (molluscicides) | 69%  63%  28%  18%  18% | 15%  90%  66%  67%  55% | 0.0000  0.0000  0.0000  0.0000  0.0000 | Positive  Positive  Positive  Positive  Positive | 0.003 | 0.550 | 0.0000 | 0.701 | 0.006 | 0.374 |
| b. What obstacles do you think will be faced when providing these interventions?   1. Lack of money 2. Lack of health facilities/hospital 3. Lack of awareness/education 4. Lack of sufficient medicine 5. Lack of infrastructure and governmental support 6. No obstacles | 15%  7%  27%  5%  -  46% | 2%  12%  74%  2%  8%  2% | 0.0000 | Positive | 0.0000 | 0.090 | 0.015 | - | 0.054 | 0.739 |
| c. Which interventions are you willing to pay for, if need be?   1. Use of clean water 2. Build toilets (sanitation) 3. Build health facility/hospital 4. Medicine 5. Education/Awareness 6. Other 7. Any future plan 8. I am not ready to contribute | 26%  28%  28%  9%  5%  -  4% | 18%  13%  -  16%  27%  -  26% | 0.4198 | Neutral+ | 0.939 | 0.058 | 0.008 | 0.005 | 0.056 | 0.465 |
| **V. Ability and contextual factors: Water contact behaviours** | | | | | | | | | | |
| 1. Why you use this water source for domestic, recreational and/or occupational needs? 2. Convenience 3. Only available source 4. Affordable 5. Privacy 6. Other | 56%  39%  2%  3%  1% | -  -  -  -  - |  |  |  |  |  |  |  |  |
| b. Will you avoid/minimise all existing contact with contaminated water if you have an alternative clean water source? | 99% | 94% | 0.0000 | Negative* | 0.134 | 0.375 | 0.657 | 0.430 | 0.456 | 0.476 |
| c. Give reasons to why you will avoid/minimise all existing contact with contaminated water   1. Since I will have clean and safe water 2. We will be disease free 3. Convenience and proximity 4. I can’t avoid water contact | 68%  31%  1%  - | -  -  -  - | - | - | - | - | - | - | - | - |
| **VI Self-regulation and contextual factors: Responsibility** | | | | | | | | | | |
| 1. Who should buy/install water infrastructure? 2. Government 3. Community fund 4. Individuals 5. All of the above 6. Other (NGOs community leader) | 67%  9%  1.5%  22%  0.5% | 76%  15%  6%  3%  - | 0.0000 | Positive | 0.112 | 0.008 | - | - | 0.00 | 0.443 |
| 1. Who should maintenance the installed water system? 2. Government 3. Community 4. Individuals/users 5. Other (NGOs, community leader) 6. All the above | 47%  41%  1%  11%  - | 18%  59%  12%  5.5%  5.5% | 0.2209 | Neutral+ | 0.318 | 0.002 | 0.196 | 0.015 | 0.016 | 0.029 |
| 1. Let’s imagine that in 5 years there is no more schistosomiasis (bilharzia), How could you achieve this? 2. Expansion of health services/centres 3. Enhancing community 4. Improve sanitation and water services 5. Avoid water contact 6. Mass drug administration 7. Increase awareness/education 8. Other | 30%  21%  14%  29%  -  -  6% | 0.5%  1%  32.5%  11%  5%  37%  13% | 0.0000 | Positive | 0.00 | 0.138 | 0.326 | 0.059 | 0.003 | 0.154 |
