# Supplementary material for: A novel theatre-based behaviour change approach for influencing community uptake of schistosomiasis control measures
Source: Parasit Vectors. 2022 Aug 25;15:301. doi: 10.1186/s13071-022-05421-5 (PMC9406251; doi:10.1186/s13071-022-05421-5)
Supplement: Supplementary file 1 — Additional file 1: Text S1. Qualitative interviews and focus group discussions topic guide and questions. Text S2. Acting for Health methodology. Table S1. Intervention workshop cohort and drama and film audience survey responses. Table S2. Emergent themes and narrative quotations from formative qualitative findings. Table S3. Quantitative questionnaire survey results for baseline and post intervention for Tanzania. Table S4. Quantitative questionnaire survey results for baseline and post intervention for Ethiopia. [file 13071_2022_5421_MOESM1_ESM.zip › Text S1.docx]

TEXT S1: Qualitative interviews and focus group discussions topic guide and questions

| **WATER INFRASTRUCTURE FOR SCHISTOSOMIASIS ENDEMIC REGIONS (WISER)** |
| --- |
| Case studies |
| **GUIDE FOR IN-DEPTH INTERVIEWS & FOCUS GROUP DISCUSSIONS** |
| **First Section of Interview/Discussions** |
| 1. Background information  Ask about the respondent’s background: age, gender, level of education, occupation, tribe, marital status, other household members in the family, how long the respondent has lived in the community, housing, method of disposal of stool, any occupant in the household who is ill/sick |
| **Second Section of Interview/Discussions** |
| 2. Assessing awareness of existence, causes, transmission, health problems, perceptions and control of schistosomiasis/ Bilharzia. |
| Assess the respondent’s knowledge by asking the following questions:   - What are the most important health problems in your community? - Have you heard about a condition called Bilharzia or schistosomiasis? - Does Bilharzia exist in your community? - Are there local names for Bilharzia? - How bad do you think the problem of Bilharzia is, compared with other health issues in the community? - What do you think causes Bilharzia? - How is Bilharzia transmitted? - Who is at most risk of contracting Bilharzia? - Who is at most risk of developing serious problems from Bilharzia? - What are the symptoms and signs of Bilharzia? - How can you prevent and control Bilharzia? - How is bilharzia generally treated in this community? (prompt for types of treatment options used whether conventional or traditional) - Have you or any family member suffered from Bilharzia? If yes, where was treatment sought and at what cost? what was the treatment given? - How does Bilharzia affect a person’s day to day activities when infected? - What have you done to prevent and control Bilharzia? - Are there any existing water contact sites/activities in this community? |
| 3. Assessing attitudes towards the various strategies for controlling schistosomiasis/ Bilharzia. |
| Ask the respondent about what he/she feels about the following methods for controlling Bilharzia and reasons. If necessary, explain what is meant by each strategy.   - Mass administration of praziquantel - Disposal of faeces in toilets/ latrines - Provision and use of safe water supplies - Minimizing contact with infested water (ponds, natural wells, streams, rivers or lake) - Biological control of snails |
| 4. Determining the readiness of the community for a WISER case study |
| Explain to the participant that at present, there is no clean/basic water infrastructure in their community sufficient for their every use, but the researchers are trying to understand how a water supply system will benefit the whole community as a possible intervention to control Bilharzia. As part of the process, it will be important to establish the readiness and willingness of the community, their full commitment to making the project a success and ensuring sustainability.  Ask the respondent the following questions (as many as time will permit):   - What is your opinion about an alternative water supply system for preventing your existing contact with water? - Will you avoid all existing contact with water if you have an alternative clean source? - Will you ensure everyone in your household avoids all existing contact with water? - Are you willing to fully participate in kind through free labour or some other way e.g. community mobilisation, if it will be a community water project? - If you had to pay for it yourself, how much would you be able and willing to contribute? - Will you agree to take ownership and contribute toward maintenance costs eventually? - What is the maximum cost you could give toward maintenance i.e., how much would you be able and willing to contribute? - Will you agree to a representative/gender inclusive community committee to be responsible for implementing and disbursement of maintenance funds? - Are there any barriers or obstacles which you think might hinder adoption of the alternative water supply system by community members and what do you think could be the best way to deal with these obstacles or barriers?   Overall (if there is still time)  What is your opinion regarding the eradication of bilharzias disease in your community?   - Are there any achievable activities you recommend to stop the transmission of bilharzia in your community? - Who should do what, when, how, where ...? |
|  |
