# Supplementary material for: A novel theatre-based behaviour change approach for influencing community uptake of schistosomiasis control measures
Source: Parasit Vectors. 2022 Aug 25;15:301. doi: 10.1186/s13071-022-05421-5 (PMC9406251; doi:10.1186/s13071-022-05421-5)
Supplement: Supplementary file 1 — Additional file 1: Text S1. Qualitative interviews and focus group discussions topic guide and questions. Text S2. Acting for Health methodology. Table S1. Intervention workshop cohort and drama and film audience survey responses. Table S2. Emergent themes and narrative quotations from formative qualitative findings. Table S3. Quantitative questionnaire survey results for baseline and post intervention for Tanzania. Table S4. Quantitative questionnaire survey results for baseline and post intervention for Ethiopia. [file 13071_2022_5421_MOESM1_ESM.zip › Text S2.docx]

**Acting for Health methodology**

The AfH methodology uses creative theatre techniques, the basis of which is an active listening paradigm that is used to explore issues and then to draw out solutions from the participants. It gives a voice to all community groups by pulling from the lived experience of the workshop participants in this study, using the different issues and perceptions that they have around water contact and schistosomiasis as well as their sense of personal responsibility, duty and agency to affect their own lives. The intervention was based around five daily sessions of three hours each which took place from 9 am to 12 noon every morning for the five days, with 16-20 workshop participants each. In the case study communities, the intervention workshop may be summarised into nine stages:

1. **a. Ice Breaking.** The first day includes activities that try to break down hierarchal barriers between the group. It is important that everyone is comfortable speaking with people of different occupations and levels of education. Vital to the cohesion of the group was the fostering of trust between each participant and trust between the group and research team.

**b. Terminology of ‘pictures’ and ‘impressions’ [1].**  Exercises through which our basic unit of expression is formed, i.e. how experiences are laid down as memories in pictures formed and impressions (emotive response elicited and associated with that picture).

**c. Imagination Exercises.** Using the pictures and impressions terminology, participants imagine breakfast scenes or other aspects of their routine life and how this can change through altering the emotive state. It introduces participants to their ability to use their mind to imagine different scenarios and leads to them to imagining a world without schistosomiasis later in the week. This was one example in a suite of exercises designed to explore imagination.

**d. Purpose [1].** This exercise illustrates to the participant that behaviour change is in part dependent upon a sense of purpose – one of the exercises revolves around a race to grab a pen, and the first person to the pen wins – however the countdown ‘3,2,1..Go’ to start is never finished and yet people start running before ‘Go’ is ever reached – they then have to describe the pictures and impressions formed in their mind behind their behaviour of running before the countdown ends – their motivations and how a clear defined purpose can illicit behaviour change. This was then used as the basis for the participants to explore a future without the disease, what it would mean for them and the steps, both personal and as a community required to reach this future.

1. **Current Knowledge.** On the first few days it is important to discuss what people in the community know, do not know and what they believe. This is discussed by asking not what the participants believe but by asking what they think people in the community believe. It ensures there is no judgement between participants, and an open space is created to find out all beliefs prevalent.
2. **Complexes [1].** ‘Complexes’ may be explained implicitly or explicitly depending on the community. Complexes were used by AfH to explore how people think and make associations. Water contact, awareness, symptoms and treatment/solutions complexes were explored. In simplest form, these are all the pictures and impressions that are in the mind of the participant regarding a word or term. After the first complex is formed, the group decides on which of the pictures or impressions to take forward in order to form its own complex, and this is represented so that a map of interplaying complexes is formed. These will be used to inform and develop character development for the play as well as provide material for the narrative flows and plotlines within each scene but also connecting the entire play. Mapping of interplaying complexes also reveals potential solutions to some of the transmission/reinfection issues faced by the participants in real life.
3. **Scenes on current understanding.** The first scenes are created on day two or three of the workshop and are designed to enable the participants to act out real life scenarios. They bring out current beliefs in the village such as becoming ill, visiting a traditional healer, misdiagnosis of the symptoms and beliefs that schistosomiasis is not a life-threatening disease. The ideas for the scenes are sourced from the discussions in stages three and four and AfH put together the scene plans. No script is used, instead improvisation to play different roles. Each scene is discussed with the rest of the group and feedback given on how realistic it is. This method enables creativity and ownership of the scene by the participants. Real life experience of treatment pathway, treatment options, disease awareness, transmission of disease and water contact behaviour were developed using this approach.
4. **Schistosomiasis Education.** On day three, the participants are educated on the accurate life cycle of schistosomiasis and clarifications are given on the mode of transmission, treatment and control/prevention methods to break the cycle. Participants ask questions and by the end all participants should have a good enough level of knowledge about schistosomiasis so that all solutions suggested in stage seven hold the same weight. This was a result of the active listening paradigm at the heart of the AFH methodology, as well as the formative and baseline findings, which had identified this knowledge gap. Hence, the decision was made to incorporate this education element, in particular transmission of the disease, into the sessions. This was also agreed on as a consensus and driven by the cohort itself.
5. **Solution Discussions.** This may occur in different forms depending on the community. For example, the participants were invited to imagine a world without schistosomiasis and think about what changes had occurred to reach that place. Or participants imagined solutions which ranged from impossible to more realistic and practical. Another example was to use the interplay between complexes formed by the groups around word and terms associated with Schistosomiasis, water contact modes etc.
6. **Final Scenes.** The solution discussions were put into a scene where the community (and audience) learn about schistosomiasis and discuss ways forward. For example, in one scene, it showed a community health worker educating other farmers on a break from harvesting. In another, a scene shows a doctor explaining the life cycle to a sick patient and their family members. A sample of scenes from a complete drama sketch is shown in Table S1 below.
7. **Play Performance.** After practising the scenes, the play was performed to the rest of the community, government officials, media and other stakeholders invited to attend. The AfH team filmed and produced an edited version of both the play as well as a ‘making of’ documentary style edit, which could both be used as an educational asset post intervention by the participants and wider community. The film of the play was shown in a community viewing centre as well as open air ‘screening’ in another village.
8. **Post play discussions.** After the performances, the government officials, audience, participants and other stakeholders were invited to discuss the way forward for the community. They were encouraged to continue these discussions as well as to use the video assets (made freely available to all through Youtube and fileshare) in the coming months to make a lasting change in the community.

A sample of scenes from a complete drama sketch is shown in Table S1 Text.

**Table S1 text**. Scenes from complete drama sketch for Kigongo village, Tanzania

|  | **Drama sketch** |
| --- | --- |
| Scene 1 | - Fishermen return from the day’s fish catch - Fish sellers meet them at lake shore to collect fish. - One fisherman has become ill with severe stomach pain and is taken home by his friends. - His mother arrives, screams and panics. - She takes him to native doctor. |
| Scene 2 | - A man who lives in Mwanza city is visiting his family in the village. - One sibling wants him to join them in harvesting rice, but he refuses because of risk of bilharzia from paddy farms. - The other sibling becomes angry and says they will not send rice to him again from the paddy fields. |
| Scene 3 | - At the lake front, domestic collection of water for washing and bathing is going on. - Some children come round to swim but are admonished by some women not to do so because of bilharzia. - One child refuses to learn and still gets in to bathe and swim at the end. |
| Scene 4 | - Sick fisherman is still unwell at home after several days. - Community Health Worker (CHW) comes around and convinces them to go to the doctor in hospital. - They go to the doctor and he diagnoses bilharzia, explains life cycle and transmission, symptoms, chronic effects etc. and encourages regular uptake of drugs and possible future use of personal protective equipment (PPE). - Prescribes praziquantel. |
| Scene 5 | - Café scene: The sick fisherman is being taken back home from hospital. They are beckoned into café by some community members, asked what is wrong and the CHW explains the sickness, what the doctor said, etc. - The community members discuss regarding many people always falling sick with similar symptoms and then they resolve to call for a community meeting. - Town crier takes the information round and invites all to attend important meeting. |
| Scene 6 | - Town hall meeting in progress. Members of the audience are encouraged to take part in this scene. - Discussions and solutions proposed by some of the community leaders and members. - Resolve to take action as a community. - Individuals are encouraged to build toilets in their homes. - Enforce it on children not to swim in lake and ponds - Decision taken for a delegation to go to the government to ask for water supply infrastructure. - Use of PPE for occupational contact especially for paddy farming is encouraged. |

**Video links:**

<https://www.youtube.com/watch?v=kxjfF0dXrQI>

<https://www.youtube.com/watch?v=h66JaTBJHUE>

**References**

1. Kogan, Sam. 'The Science of Acting'. Routledge, 1st edition. 2009.
