# Supplementary material for: A novel theatre-based behaviour change approach for influencing community uptake of schistosomiasis control measures
Source: Parasit Vectors. 2022 Aug 25;15:301. doi: 10.1186/s13071-022-05421-5 (PMC9406251; doi:10.1186/s13071-022-05421-5)
Supplement: Supplementary file 2 — Additional file 2: Figure S1. Some selected images from intervention workshops. Figure S2. Flyer with life cycle, transmission and control in Tanzania [file 13071_2022_5421_MOESM2_ESM.zip › Figure S1.pdf]

Some selected images from intervention workshops

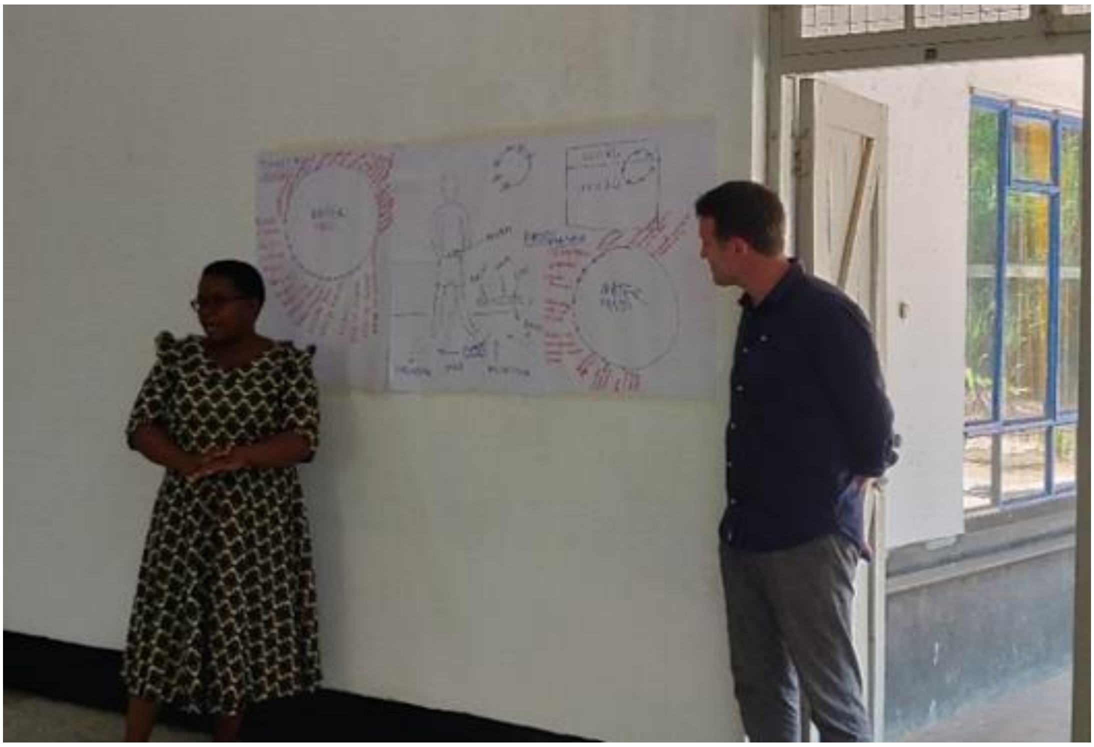

Fig S1 (a): Developing Tanzania cohort complex around bilharzia

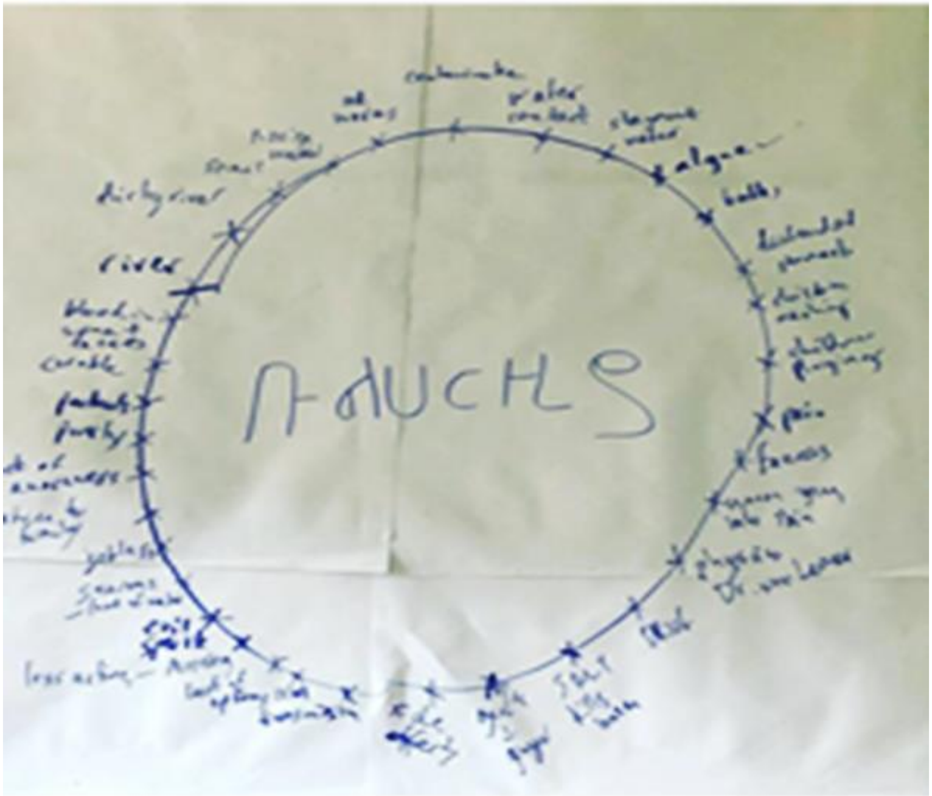

Fig S1 (b): Ethiopian cohort complex around bilharzia

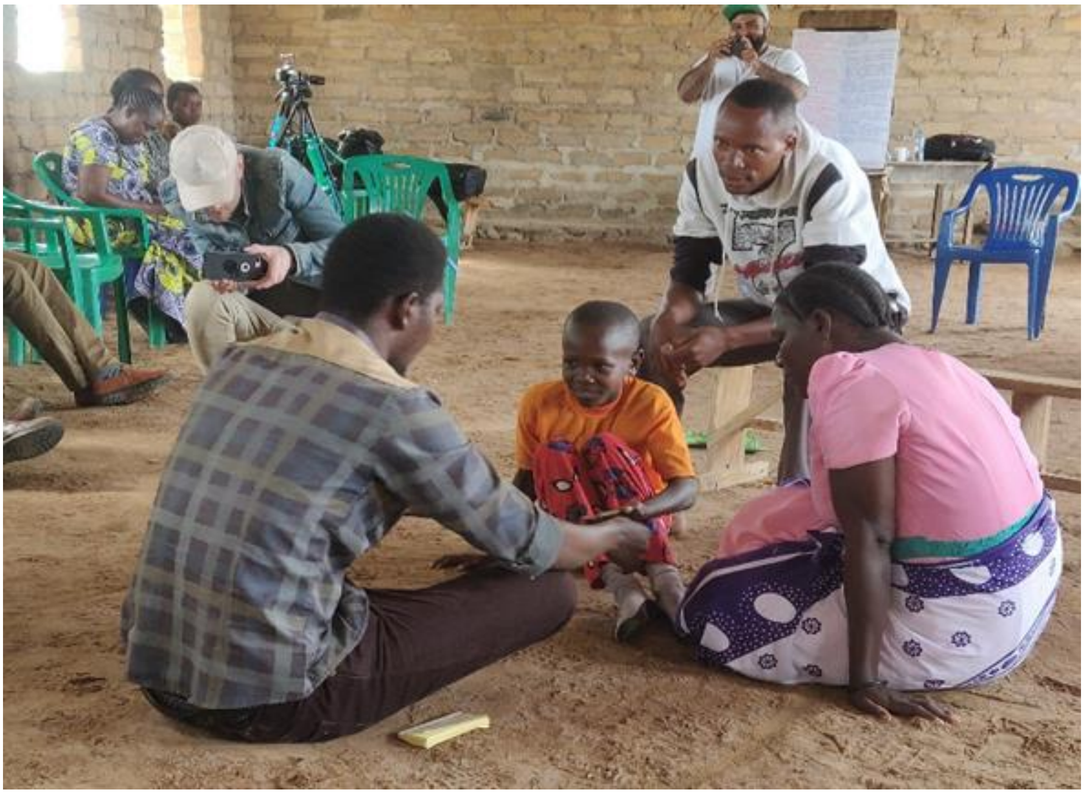

Fig S1 (c): Traditional medicine man scene in Tanzania

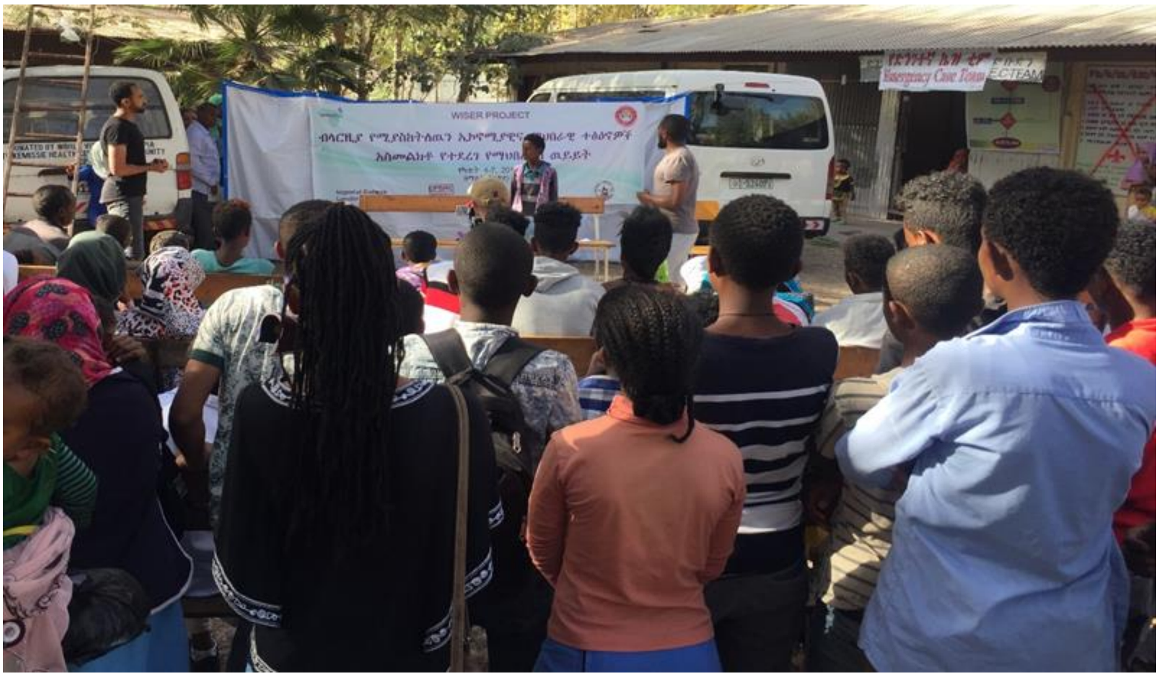

Fig S1 (d): Play performance in Ethiopia

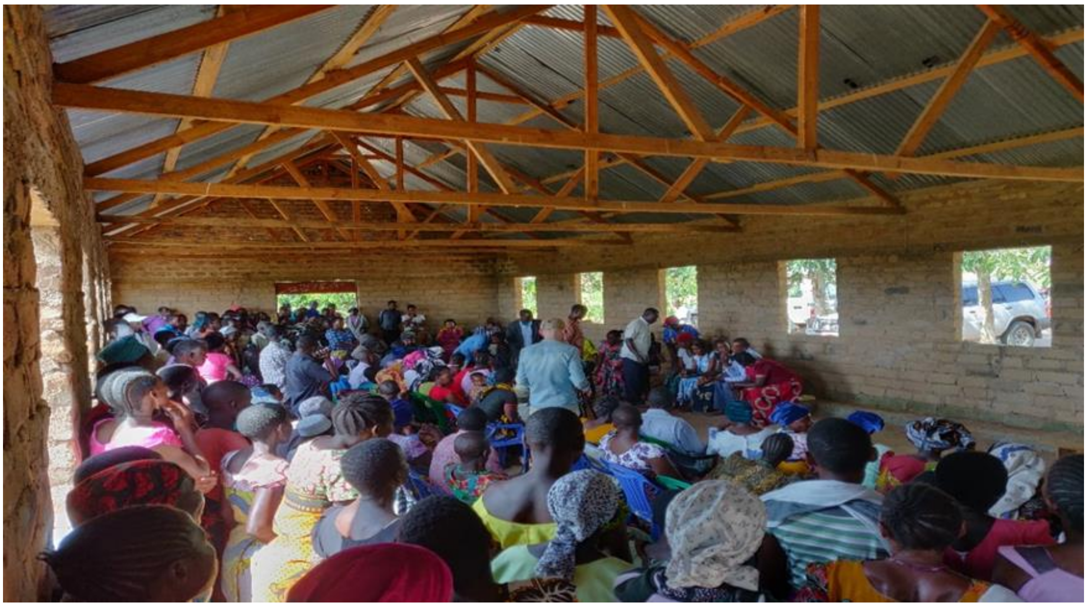

Fig S1 (e): Play performance in Tanzania: Mwakalima

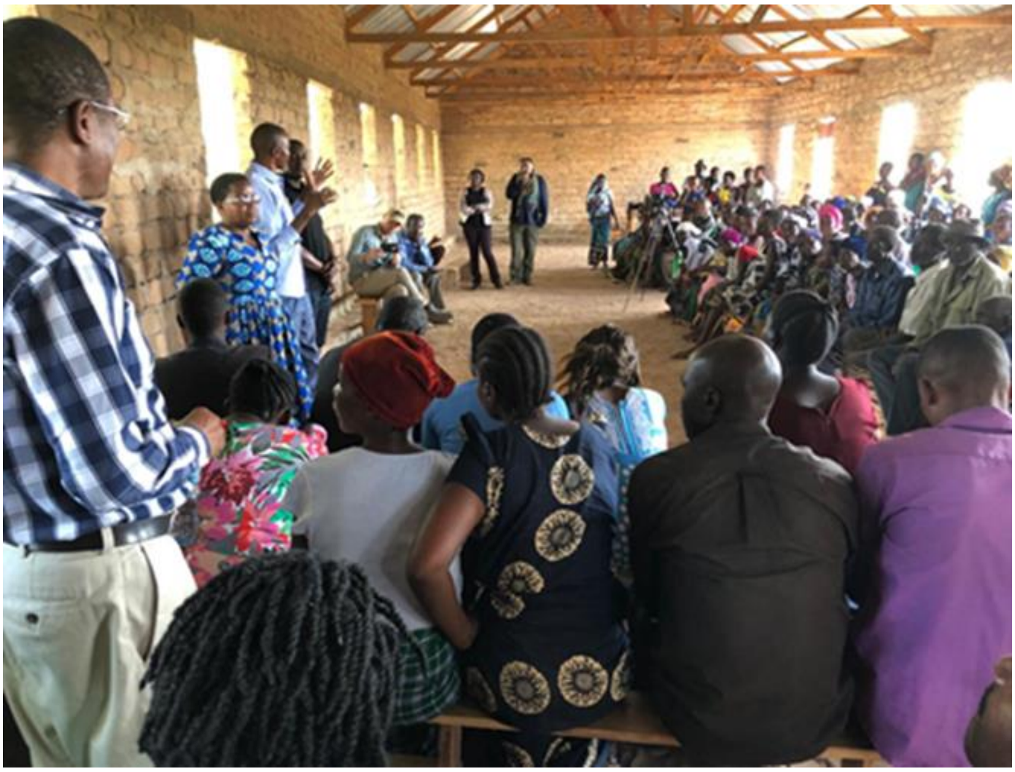

Fig S1 (f): Community leaders talking to audience after play performance in Tanzania

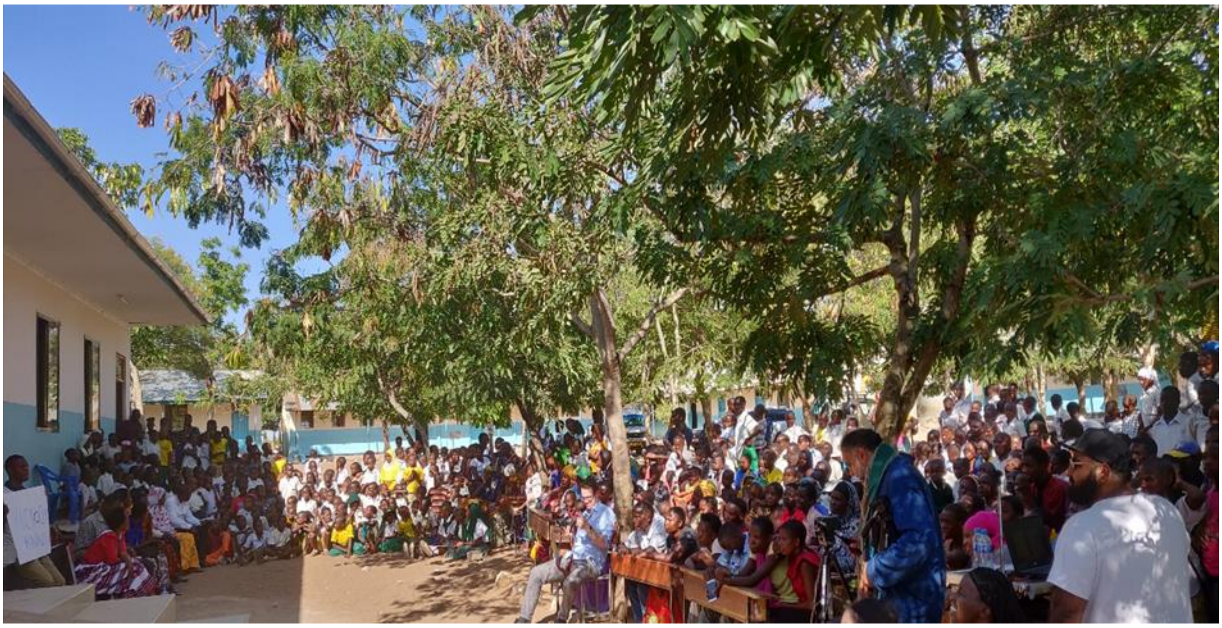

Fig S1 (g): Play performance in Tanzania: Kigongo

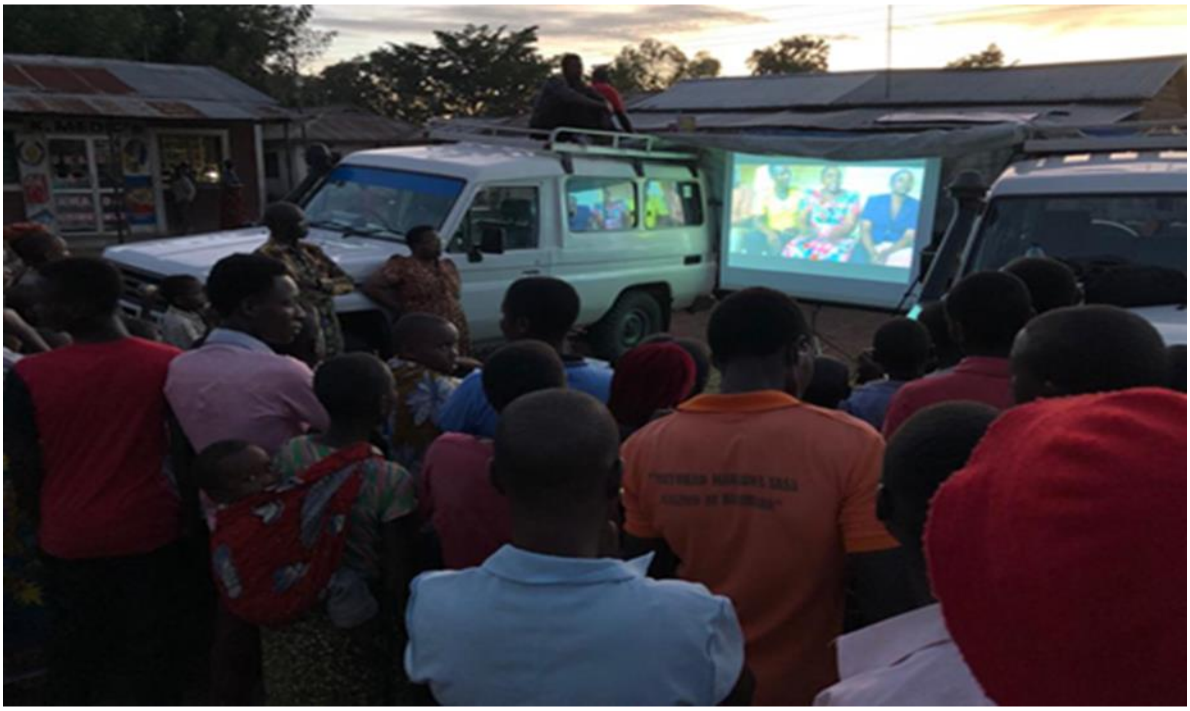

Fig S1 (h): Open air ‘screening’ of film of play in Tanzania: Nyangholongo
