# Supplementary material for: A novel theatre-based behaviour change approach for influencing community uptake of schistosomiasis control measures
Source: Parasit Vectors. 2022 Aug 25;15:301. doi: 10.1186/s13071-022-05421-5 (PMC9406251; doi:10.1186/s13071-022-05421-5)
Supplement: Supplementary file 2 — Additional file 2: Figure S1. Some selected images from intervention workshops. Figure S2. Flyer with life cycle, transmission and control in Tanzania [file 13071_2022_5421_MOESM2_ESM.zip › Figure S2.pdf]

Dalili za ugonjwa wa kichocho

Kwa ujumla, dalili za ugonjwa wa kichocho ni pamoja na homa, mwili kuishiwa nguvu, kupoteza hamu ya kula na maumivu ya viungo vya mwili.

Dalili za kichocho cha tumbo ni:

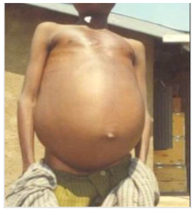

- Maumivu ya tumbo
- Kuharisha damu
- Ini na bandama kuvimba
- Kuvimba tumbo
- Upungufu wa damu

Dalili za kichocho cha mkojo ni:

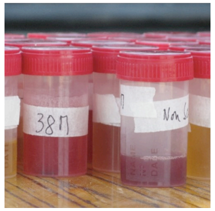

1. Kukojoa damu
2. Maumivu wakati wa kukojoa
3. Maumivu sehemu ya chini ya tumbo
4. Upungufu wa damu

Madhara yatokanayo na ugonjwa wa kichocho

Madhara yafuatayo yanaweza kutokea baada ya mwathirika kukaa na ugonjwa wa kichocho kwa muda mrefu bila matibabu:

- Kuharibika kwa ini, bandama na viungo vingine vya ndani
- Kuharibika kwa kibofu cha mkojo
- Kudumaa kwa mwili, akili na uwezo mdogo wa kujifunza kwa watoto wa shule
- Saratani ya kibofu cha mkojo
- Ugumba
- Utapiamlo kwa watoto
- Uwezo mdogo wa kufanya kazi za uzalishaji mali kwa watu wazima

Kichocho kinatibiwaje?

Kichocho hutibiwa kwa dawa ya vidonge aina ya Praziquantel. Dawa hii inapatikana hospitalini na katika maduka ya dawa.

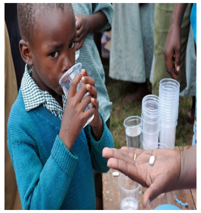

Picha: Mtoto akinywa dawa ya praziquantel

Pia dawa hii hutolewa mara moja kila mwaka kwa watoto wenye umri wa kwenda shule kwa sababu kundi hili la jamii liko katika hatari kubwa zaidi ya kupata maambuki ya ugonjwa wa kichocho. Idadi ya vidonge vya praziquantel hutolewa kwa kuzingatia uzito au urefu wa mgonjwa.

Ni muhimu kupima na kurudia tena matibabu kwa sababu maambukizi yanaweza kutokea tena baada ya matibabu ya awali.

Njia za kujikinga na ugonjwa wa kichocho

- Kutumia vyoo wakati wote kwa ajili ya haja kubwa na haja ndogo.
- Kuepuka kujisaidia ovyo kandokando ya njia, katika mazingira tunayoishi, kwenye vyanzo vya maji au vichakani.
- Kuvaa mavazi ya kujikinga kama buti wakati wa kazi za kilimo, uvuvi, ulishaji mifugo n.k.
- Kutumia vyanzo safi na salama vya maji kwa ajili ya mahitaji yote ya nyumbani.
- Kupata elimu sahihi juu ya ugonjwa wa kichocho na elimu ya afya kwa ujumla.
- Kwenda hospitali kupima na kupata matibabu pindi unapoona dalili za ugonjwa wa kichocho.

Hitimisho

Ugonjwa wa kichocho ni tatizo kubwa la kiafya nchini Tanzania na katika nchi zingine za ukanda wa joto. Jitihada za kupambana na ugonjwa huu zinaendelea na mafanikio yamepatikana. Hata hivyo tatizo la ugonjwa wa kichocho bado ni kubwa hali ambayo inaashiria kwamba jitihada zaidi za kupambana na kichocho zinahitajika.

Njia za kudhibiti ugonjwa wa kichocho zinazoshirikisha wadau mbalimbali ikiwa ni pamoja na watafiti, viongozi, wahudumu wa sekta ya afya na wanajamii kwa ujumla zinaweza kuleta mafanikio katika kudhibiti na hatimaye kutokomeza kabisa ugonjwa wa kichocho.

Taasisi ya Taifa ya utafiti wa Magomjwa ya Binadamu (NIMR)
Kituo cha Mwanza

UGONJWA WA KICHOCHO

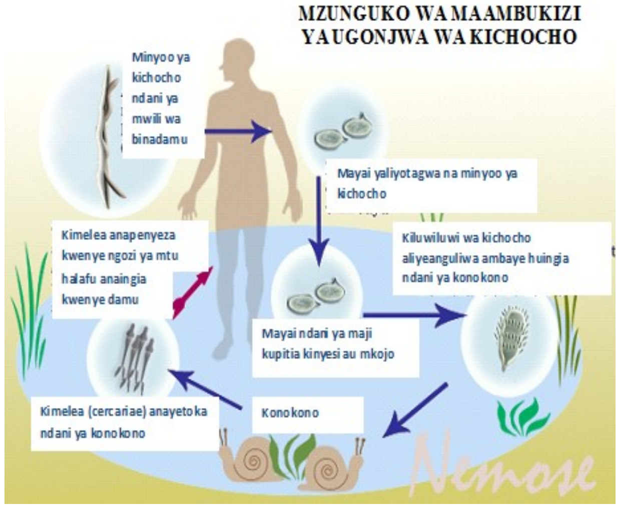

Kipeperushi hiki kimeandaliwa na taasisi ya taifa ya utafiti wa magonjwa ya binadamu (NIMR), kituo cha Mwanza, kwa hisani ya mradi wa WISER

Kichocho ni nini?

Kichocho ni ugonjwa wa kuambukiza unaosababishwa na minyoo aina ya *Schistosoma.* Maambukizi hutokea wakati binadamu anapokanyaga au kugusa maji yenye vimelea vya ugonjwa wa kichocho. Aidha vimelea vya ugonjwa wa kichocho husambazwa na konokono wanaoishi na kuzaliana katika vyanzo mbalimbali vya maji kama madimbwi, mito, maziwa na visima vya asili.

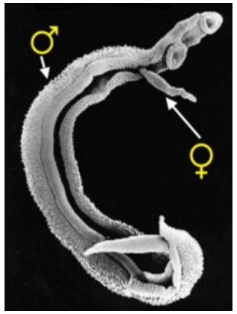

Picha: Mnyoo wa kichocho (Schistosoma)

Aina za ugonjwa wa kichocho

Kuna aina kuu mbili za minyoo inayosababisha ugonjwa wa kichoo inayopatikana katika bara la Afrika ikiwa ni pamoja na Tanzania. Aina hizi ni:

Kichocho cha tumbo: Aina hii ya kichocho husababishwa na minyoo inayoitwa ***Schistosoma mansoni***

Kichocho cha mkojo: Aina hii ya kichocho husababishwa na minyoo inayoitwa ***Schistosoma haematobium***

Aina zote mbili za minyoo ya kichocho zinafanana sana kimaumbile na si rahisi kuzitofautisha kwa macho. Hata hivyo tofauti yake huonekana kwa kuangalia mayai ya minyoo hii kwenye darubini au njia zingine za kitaalamu katika maabara.

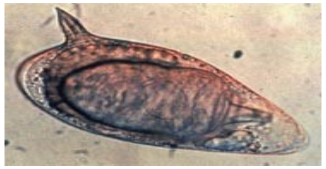

Picha: Yai la mnyoo wa kichocho cha tumbo

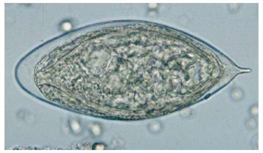

Picha:Yai la mnyoo wa kichocho cha mkojo

Ukubwa wa tatizo la ugonjwa wa kichocho

Kichocho ni ugonjwa ulioenea sana katika ukanda wa nchi za joto zinazojulikana kitaalamu kama nchi za kitropiki.

Kichocho kinahesabika kama ugonjwa wa tatu kwa umuhimu kutokana na kiwango cha madhara ya kiafya unayosababisha baada ya ugonjwa wa malaria na minyoo mingine ya tumbo.

Ugonjwa wa kichocho unapatikana katika jumla ya nchi 76 kati ya nchi 195 duniani kote lakini zaidi ya asilimia 90 ya watu wote walioathirika na ugonjwa wa kichocho wanaishi katika nchi za bara la Afrika kusini mwa jangwa la Sahara.

Tanzania ni mojawapo ya nchi zilizoathirika sana na ugonjwa wa kichocho na inaaminika kuwa ni nchi ya pili kwa kiwango cha maambukizi baada ya nchi ya Nigeria.

Katika Tanzania, kichocho kimeenea nchi nzima, lakini kiwango cha juu zaidi cha maambukizi kiko katika ukanda wa Ziwa Victoria pamoja na ukanda wa bahari ya Hindi. Katika maeneo haya, kiwango cha maambukizi hufikia hadi asilimia 100 katika baadhi ya maeneo.

Nani yupo katika hatari kubwa zaidi ya kupata maambukizi ya ugonjwa wa kichocho?

Watu waishio karibu na vyanzo vya maji (kama maziwa, mito, madimbwi, mabwawa, visima vya asili) vyenye maji yenye vimelea vya kichocho. Hata hivyo makundi yafuatayo yako katika hatari kubwa zaidi ya kupata maambukizi:

- Watoto wenye umri wa kwenda shule: Kwa sababu ya tabia ya kuchezea maji na matope na kutozingatia tabia ya usafi
- Wavuvi: Kwa sababu ya shughuli zao za uvuvi hukanyaga na kugusa maji mara kwa mara
- Wakulima wa mpunga au mashamba makubwa ya umwagiliaji: Kwa sababu ya shughuli zao za ukulima hukanyaga na kugusa maji mara kwa mara.

Binadamu huambukizwaje ugonjwa wa kichocho?

Maambukizi ya ugonjwa wa kichocho hutokea katika maeneo yenye maji kwa mfano ziwani, mtoni, madimbwi, majaruba, mabwawa na visima vya asili. Binadamu huambukizwa ugonjwa wa kichocho pale anapokanyaga maji yaliyo na vimelea vya kichocho. Vimelea hivi hupenyeza kwenye ngozi ya mhusika na kuingia kwenye mishipa ya damu ambapo husambazwa hadi kwenye utumbo au kibofu cha mkojo ambako vimelea wa ugonjwa wa kichocho huzaliana na kusababisha madhara.

Maji yanapata vimelea vya ugonjwa wa kichocho pale yanapochafuliwa na kinyesi au mkojo wa mtu mwenye ugonjwa wa kichocho.

Kinyesi au mkojo wa mtu mwenye ugonjwa wa kichocho hubeba mayai ya vimelea wa ugonjwa wa kichocho. Kinyesi au mkojo huu unapopata nafasi ya kuingia kwenye maji, mayai haya huanguliwa na kutoa viluwiluwi vya vimelea vya ugonjwa wa kichocho.

Viluwiluwi hivi huwaingia konokono walioko kwenye maji. Ndani ya konokono viluwiluwi hukua na kutoa vidudu vingine viitwavyo cercariae. Cercariae hutoka ndani ya konokono na kuingia kwenye maji. Pale ambapo binadamu atakanyaga maji yenye cercariae wa kichocho, cercariae humwingia mtu huyo kupitia kwenye ngozi yake na kukamilisha mzunguko wa ukuaji wa vimelea wa kichocho.
